# The Influence of Innate Immunity, Adaptive Immunity and Diet on Intestinal Microbiota Following *Trichuris muris* Infection

**DOI:** 10.1111/pim.70060

**Published:** 2026-02-02

**Authors:** Bridgious Walusimbi, Kelly S. Hayes, Melissa A. E. Lawson, Seona Thompson, Allison J. Bancroft, Alison M. Elliott, Richard K. Grencis

**Affiliations:** ^1^ Immunomodulation and Vaccines Group, Vaccine Research Theme, Medical Research Council Uganda Virus Research Institute and London School of Hygiene and Tropical Medicine (MRC/UVRI & LSHTM) Uganda Research Unit Entebbe Uganda; ^2^ Department of Immunology & Molecular Biology School of Biomedical Science, College of Health Sciences, Makerere University Kampala Uganda; ^3^ Department of Clinical Research London School of Hygiene and Tropical Medicine London UK; ^4^ Faculty of Biology Medicine and Health, University of Manchester Manchester UK

## Abstract

*Trichuris trichiura* infects nearly 500 million people worldwide, causing intestinal inflammation, malnutrition, and growth impairment, particularly in children from low‐resource settings. While host immunity is central to parasite clearance, diet and the gut microbiota may also modulate infection. Using the *Trichuris muris* model, we examined how immune competence and diet interact to influence worm burden, antibody responses, and gut microbiota composition. Wild‐type (WT), RAG1‐deficient (lacking adaptive immunity), and RAG1/*γ*c‐deficient (lacking both adaptive and innate lymphoid immunity) mice were fed either a normal diet (ND) or high‐fat diet (HFD) and infected with a low dose of *T. muris*. WT mice on ND developed chronic infection with strong IgG2a/c responses, consistent with Th1‐biased immunity. In contrast, WT mice on HFD achieved near‐complete parasite clearance, accompanied by elevated IgG1 and reduced IgG2a/c titres, indicating diet‐induced Th2 bias. In RAG1‐ and RAG1/*γ*c‐deficient mice, infection persisted under a normal diet but worm burdens were partially reduced on HFD, indicating that diet enhances parasite control through immune‐independent, possibly microbiota‐mediated pathways. Microbiota clustered by genotype and diet, with HFD‐associated enrichment of *Bacteroides*, *Parabacteroides*, and *Blautia*. These findings demonstrate that diet and immune status jointly shape helminth susceptibility through coordinated effects on host immunity and the gut microbiota.

## 1 Introduction

Parasitic helminths such as *Trichuris trichiura* remain a major public health concern in low‐ and middle‐income countries (LMICs), where they affect over 500 million individuals and contribute substantially to the global burden of neglected tropical diseases [1]. Chronic infection, particularly in paediatric populations, is associated with impaired linear growth, micronutrient deficiencies, anaemia, and persistent intestinal inflammation [2, 3, 4]. These parasitic diseases now increasingly intersect with emergent health challenges in LMICs, where rapid urbanisation and globalisation have driven widespread shifts in dietary patterns, most notably increased consumption of ultra‐processed, high‐fat foods [5]. This transition has coincided with a growing burden of metabolic dysfunction, creating a syndemic landscape in which helminth infections and diet‐induced inflammation coexist within the same host [6, 7].

While the immunological mechanisms of helminth clearance, especially the requirement for CD4^+^ Th2‐polarised responses, are well characterised [8, 9, 10], far less is known about how dietary exposures and immune competence jointly modulate host susceptibility, anti‐helminth immunity, and gut microbial ecology. Diets rich in saturated fats have been shown to impair intestinal barrier function and drive low‐grade systemic inflammation, potentially attenuating effective immune responses to helminth infection and altering microbial composition [11, 12]. Moreover, genetic ablation of adaptive or innate immune pathways, including T, B, and innate lymphoid cells (ILCs), can lead to marked dysbiosis [13, 14, 15]. However, the extent to which these immunological deficits interact with dietary factors to shape helminth–microbiota dynamics remains poorly defined.

To begin to address this gap in knowledge, we used a tractable murine model of *Trichuris muris* infection, a close immunological and ecological analogue of human *T. trichiura*, to delineate the individual and combined contributions of host immunity and diet in shaping gut–parasite–microbiota interactions. We utilised three genetically distinct mouse models: wild‐type (WT), *RAG1*‐deficient (*RAG1* ^−^/^−^; lacking T and B cells), and *RAG1/γc*‐deficient (*RAG1* ^−^/^−^ *γc* ^−^/^−^; lacking T, B, and ILCs) mice, and exposed them to either a normal diet (ND) or high fat diet (HFD) prior to infection. Parasite burden, intestinal microbial composition, and antigen‐specific IgG1 and IgG2a/c titres were assessed following infection.

## 2 Methods

### 2.1 Animal Experiments and Dietary Intervention

Three genetically distinct mouse models were used in this study to investigate the effects of innate immunity, adaptive immunity and diet on gut microbiota and clearance of *Trichuris muris* infection. These included WT C57BL/6j immunocompetent mice, C57BL/6j *RAG1* ^−/−^ deficient (RAG1 KO) mice lacking T and B lymphocytes, and C57BL/6j *RAG1* ^*−/−*^ */γc* ^*−/−*^ deficient (RAG*γ*c) mice deficient in T cells, B cells, and innate lymphoid cells (ILCs). All mice were bred and housed under specific pathogen‐free (SPF) conditions at University of Manchester, with *ad libitum* access to food (normal chow or high fat) and water. Animals were maintained in individually ventilated cages with 65% humidity at 21°C°–23°C. Experiments were performed under the regulations of the Home Office Scientific Procedures Act (1986), (Licence P043A082) and were subject to local ethical review by the University of Manchester Animal Welfare and Ethical Review Body (AWERB) and followed ARRIVE 2.0 guidelines. Male and female mice were used. Mice were randomly assigned into cages and age/sex matched in experimental groups as far as possible. Weight and body condition were monitored throughout the experimental period and were comparable between groups within dietary regime.

**FIGURE 1:**
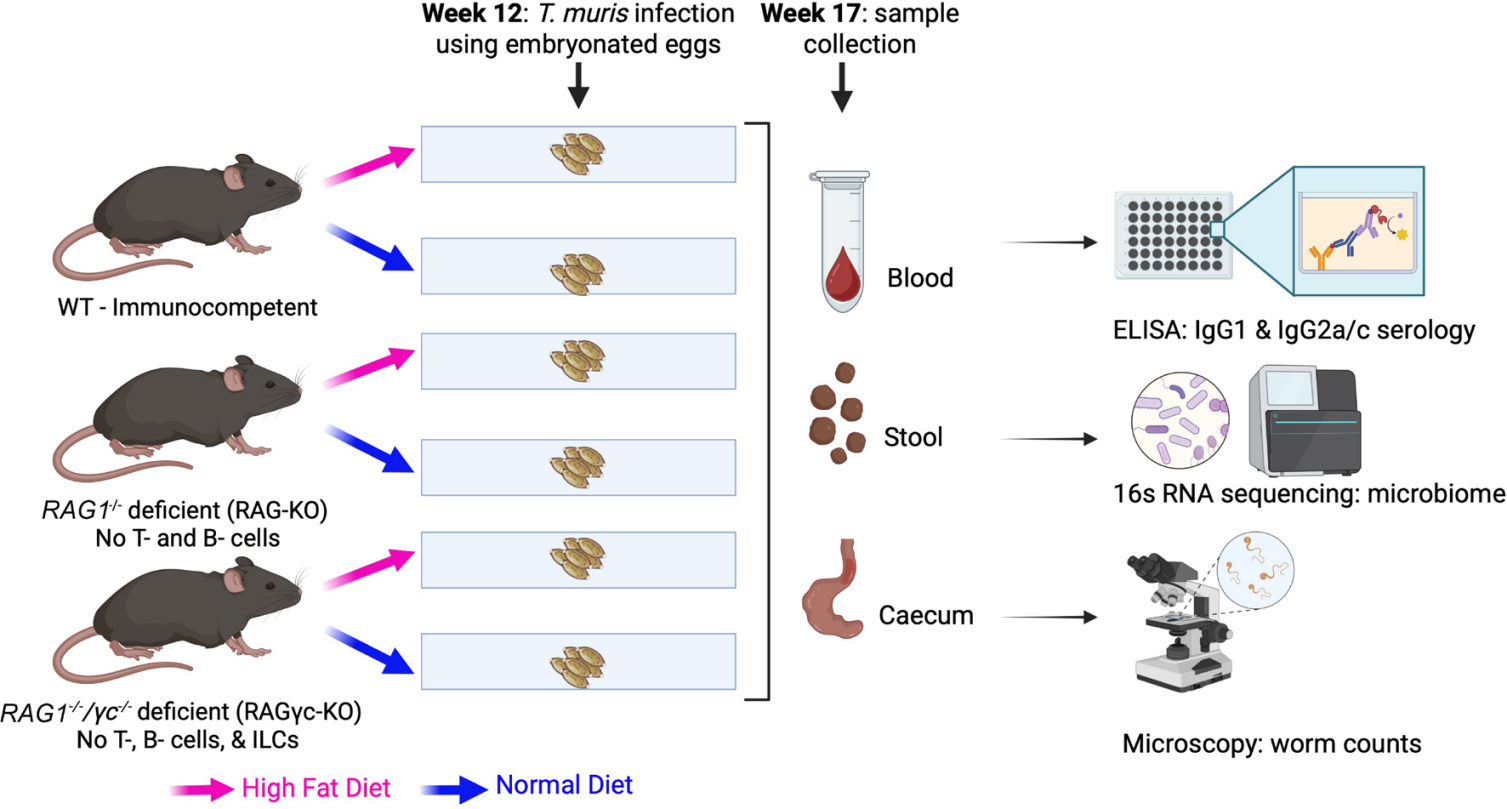
Flowchart of the research experiment drawn using icons from BioRender.

At 5 weeks of age, mice were randomly assigned to receive either a standard chow diet (ND) or a high‐fat diet (HFD) (DIO Rodent Purified Diet containing 60% energy from fat, blue, irradiated; Research Diets Inc.). Diets were maintained throughout the experiment. At week 12, all mice were orally infected with 25 embryonated *T. muris* eggs. Animals were monitored regularly and euthanized at week 17, corresponding to day 42 post‐infection when the infection had reached patency. At necropsy, caecal content, faecal pellets, and serum were collected and immediately stored at −80°C for subsequent experiments and downstream analyses as shown in Figure 1.

### 2.2 Worm Burden Quantification

Worm burdens were quantified blindly by carefully dissecting the caeca, opening them longitudinally in phosphate‐buffered saline (PBS), and counting *T. muris* worms under a dissecting microscope.

### 2.3 Parasite‐Specific Antibody ELISA

Serum levels of *T. muris*–specific IgG1 and IgG2a/c were measured by enzyme‐linked immunosorbent assay as previously described [16]. Plates were coated with *T. muris* excretory/secretory antigen, and bound antibodies were detected using biotinylated anti‐mouse IgG1 or IgG2a/c followed by streptavidin–horseradish peroxidase and 2,2′‐azino‐bis(3‐ethylbenzothiazoline‐6‐sulfonic acid) substrate. Additional methodological details are provided in the Supporting Information.

### 2.4 Microbiome Sample Preparation and Sequencing

Microbial DNA was extracted from faecal samples using the QIAamp DNA Stool Mini Kit (Qiagen). The V3–V4 hypervariable region of the 16S rRNA gene was amplified using the Illumina 16S Metagenomic Sequencing Library Preparation protocol [17].

Sequencing was done on an Illumina MiSeq platform using a 2 × 300 bp paired‐end MiSeq Reagent Kit v3 (600‐cycle) at the Bioinformatics Core Facility, University of Manchester.

Caecal contents were subjected to shotgun metagenomic sequencing Transnetyx (UK; https://www.transnetyx.com) using Illumina short‐read technology. Additional methodological details are provided in the Supporting Information.

### 2.5 Microbiome Bioinformatics Processing

Raw demultiplexed FASTQ files were processed in QIIME2 (v2023.2). Primers were trimmed using Cutadapt (v4.4), and reads were denoised, merged, and chimera‐checked with DADA2 to generate amplicon sequence variants (ASVs). Taxonomy was assigned using a Naive Bayes classifier trained on the Greengenes 13.8 database. Samples were rarefied to 20,000 sequences. Alpha diversity (Shannon index, observed richness) and beta diversity (Bray–Curtis dissimilarity) were computed and visualised using PCoA. Group differences in community composition were tested by PERMANOVA (999 permutations). Differential abundance analysis was performed in R using DESeq2 within the phyloseq framework, applying Wald tests and Benjamini–Hochberg FDR correction (*q* < 0.05). Significantly altered taxa (|log_2_FC| ≥ 1) were visualised using volcano and waterfall plots. Additional methodological details are provided in the Supporting Information.

### 2.6 Statistical Analysis

Statistical analyses were performed using R (version 4.1.3). Group comparisons for worm burden and alpha diversity indices were conducted using one‐way ANOVA or Kruskal‐Wallis tests, depending on data distribution. Two‐way ANOVA was used to compare parasite‐specific antibody responses across serum dilution series. For all tests, a *p* < 0.05 was considered statistically significant.

## 3 Results

We analysed faecal samples from a total of 49 mice, stratified by immune genotype (WT, RAG1‐deficient [RAG‐KO], and *RAG1* ^*−/−*^ */γc* ^*−/−*^ deficient [RAG*γ*c]), dietary exposure (normal chow [ND] vs. high‐fat diet [HFD]), and *T. muris* infection status (infected vs. naïve), as shown in Table 1. Among infected groups, mice included WT_N (*n* = 7), WT_H (*n* = 4), RAG_N (*n* = 7), RAG_H (*n* = 7), RAG*γ*c_N (*n* = 7), and RAG*γ*c_H (*n* = 8). In addition, uninfected (naive) control groups comprised WT_N (*n* = 5) and RAG_N (*n* = 4) mice maintained on a ND.

**TABLE 1:** Mouse group characteristics by genetic background, diet, and infection status.

Immune phenotype	Diet	Group (DIET_mousetype)	Number of mice	*T. muris* infection status
WT	ND	WT_N	7	Infected
WT	HFD	WT_H	4	Infected
RAG‐KO	ND	RAG_N	7	Infected
RAG‐KO	HFD	RAG_H	7	Infected
RAG*γ*c‐KO	ND	RAG*γ*c_N	7	Infected
RAG*γ*c‐KO	HFD	RAG*γ*c_H	8	Infected
WT	ND	WT_N	5	Naive
RAG‐KO	ND	RAG_N	4	Naive

Abbreviations: HFD, high fat diet; ND, normal diet; RAG‐KO, RAG knockout mice (no adaptive immunity); RAG*γ*c‐KO, RAG1/*γ*c‐deficient mice (deficient in innate and adaptive immunity); WT, intact immunity.

### 3.1 Host Immune Competence and Diet Influence *Trichuris muris* Worm Burden

**FIGURE 2:**
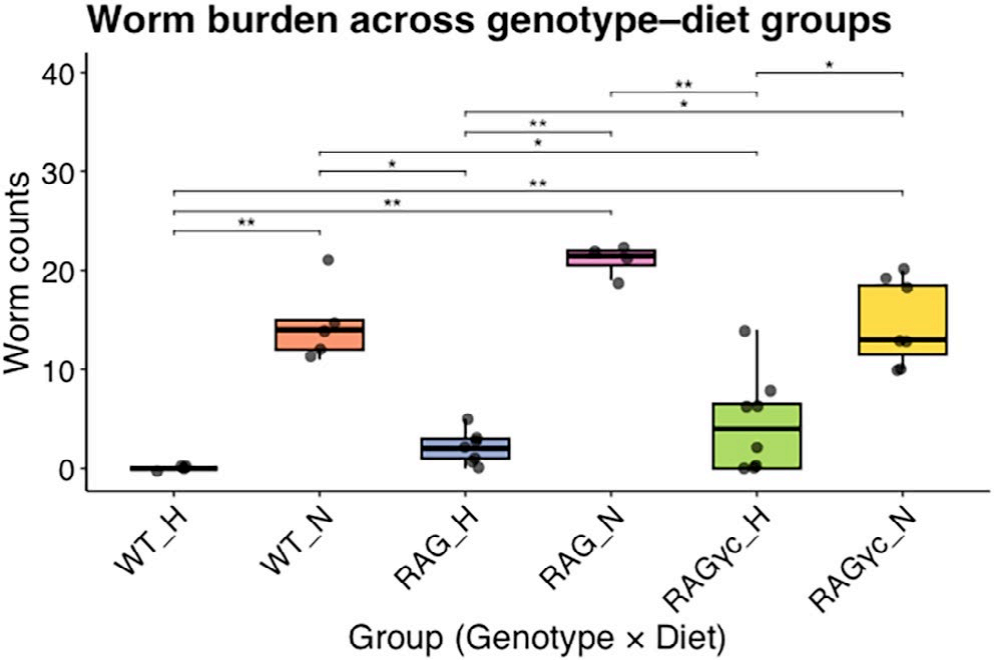
Worm burden across mouse genotypes and dietary groups following *T. muris* infection. Boxplots show intestinal worm counts in infected wild‐type (WT), RAG‐deficient (RAG), and RAG*γ*c‐deficient mice fed either a normal diet (ND) or a high‐fat diet (HFD). Consistent with impaired type‐2 immunity, RAG‐deficient mice on an ND exhibited the highest worm burdens, whereas WT mice on an HFD showed complete parasite clearance. Overall group differences were significant (Kruskal–Wallis test, *p* = 5.8 × 10^−5^). Post hoc Dunn tests (Benjamini–Hochberg correction) identified significant pairwise differences, including WT_H versus WT_N (*p*_a_d_j_ = 0.0060), WT_H versus RAG_N (*p*_a_d_j_ = 0.0013), WT_H versus RAG*γ*c_N (*p*_a_d_j_ = 0.0051), WT_N versus RAG_H (*p*_a_d_j_ = 0.0307), WT_N versus RAG*γ*c_H (*p*_a_d_j_ = 0.0435), RAG_H versus RAG_N (*p*_a_d_j_ = 0.0049), RAG_H versus RAG*γ*c_N (*p*_a_d_j_ = 0.0262), RAG_N versus RAG*γ*c_H (*p*_a_d_j_ = 0.0051), and RAG*γ*c_H versus RAG*γ*c_N (*p*_a_d_j_ = 0.0378). These comparisons clarify both diet‐specific effects within genotypes and genotype‐dependent effects within diets.

To evaluate how host immune competence and dietary environment influence parasite clearance, we assessed the impact of host immune genotype and diet on *T. muris* worm burden across different mouse models (Figure 2). Under a normal diet (ND), all three genotypes—wild‐type (WT_N), RAG‐KO (RAG_N), and RAG*γ*c‐KO (RAG*γ*c_N)—harboured low worm burdens, with no significant differences between groups despite slight variation in mean worm counts. This indicates that under normal dietary conditions and low dose infection, host immune status alone does not strongly influence the establishment of chronic infection.

In contrast, worm burdens were markedly reduced under HFD. WT mice on HFD (WT_H) showed complete parasite clearance across all individuals while RAG‐KO and RAG*γ*c_N KO mice exhibited substantial but incomplete clearance of worms. A Kruskal‐Wallis test confirmed significant differences in worm burdens across groups (*p* = 5.8 × 10^−5^). These results demonstrate that the interaction of host immune competence and diet is essential for parasite clearance, with HFD exposure strongly reducing worm burden across genotypes and immunity, but complete parasite clearance occurring only in immune‐competent mice.

Post hoc Dunn tests (Benjamini–Hochberg correction) identified significant reductions in worm burden when comparing WT_H to its ND counterpart (WT_N) and to lymphocyte‐deficient groups (RAG_N and RAG*γ*c_N), highlighting a strong diet effect within the WT background. Within the RAG and RAG*γ*c genotypes, HFD also significantly reduced worm burden (e.g., RAG_H vs. RAG_N; RAG*γ*c_H vs. RAG*γ*c_N). Additionally, several significant genotype‐dependent differences emerged within diet strata (e.g., WT_N vs. RAG_H; WT_N vs. RAG*γ*c_H).

Together, these analyses demonstrate that both immune competence and diet shape parasite clearance, with HFD exerting a broad suppressive effect on worm burden across genotypes, but complete parasite elimination occurring only in immune‐competent WT mice on HFD. Full pairwise Dunn test results are provided in Table S1.

### 3.2 Parasite‐Specific IgG1 and IgG2a/c Responses Show Diet‐Dependent Immune Polarisation

**FIGURE 3:**
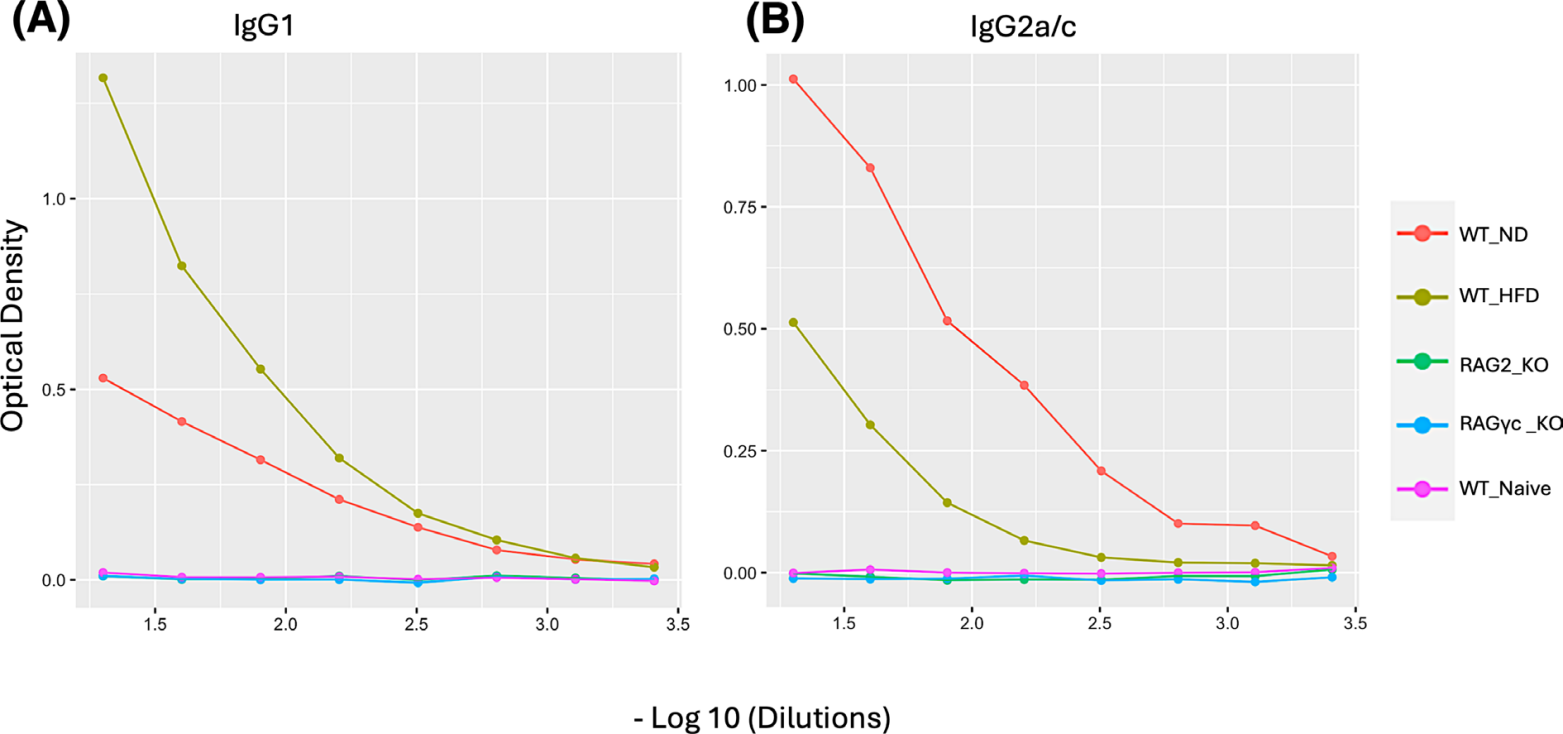
Parasite‐specific IgG1 and IgG2a/c antibody responses across immune genotypes and dietary exposures. (A) Optical density (OD) readings for parasite‐specific IgG1 across serial serum dilutions in wild‐type mice on ND (WT_ND), WT mice on HFD (WT_HFD), RAG_KO, RAG*γ*c_KO, and naïve wild‐type controls. (B) Corresponding OD readings for parasite‐specific IgG2a/c across the same groups. WT_HFD mice exhibited the strongest IgG1 responses. WT_ND mice showed highest IgG2a/c antibody titres, while RAG1_KO and RAG*γ*c _KO mice, which lack adaptive immunity, failed to mount detectable responses. Naïve mice served as uninfected controls.

Given the differences in worm burden, we next assessed parasite‐specific IgG1 and IgG2a/c responses as proxy serological markers of Type 2 and Type 1 immunity respectively (Figure 3). This allowed us to determine whether impaired parasite expulsion was associated with altered antibody production across genotypes and dietary exposures. As expected, WT mice on a normal diet (WT_ND) with low‐dose chronic infection developed a mixed response, characterised by detectable IgG1 and a predominant IgG2a/c signal, consistent with Type 1‐skewed immunity in the context of persistent infection. In contrast, WT mice on a high‐fat diet (WT_H) that expelled their parasites mounted a strong IgG1 response with only weak IgG2a/c production, reflecting a shift toward Type 2 immunity associated with effective clearance.

As expected, RAG‐KO and RAG/*γ*c‐deficient mice failed to generate detectable parasite‐specific antibodies, confirming their lack of adaptive immunity. Naive mice showed no measurable parasite‐specific antibody responses. These results demonstrate that dietary environment modulates the balance of Type 1 versus Type 2 immunity in WT mice, in line with the patterns reported by Fujinka et al. [16].

### 3.3 Relative Abundance of the Gut Microbiota Across Mouse Genotypes

**FIGURE 4:**
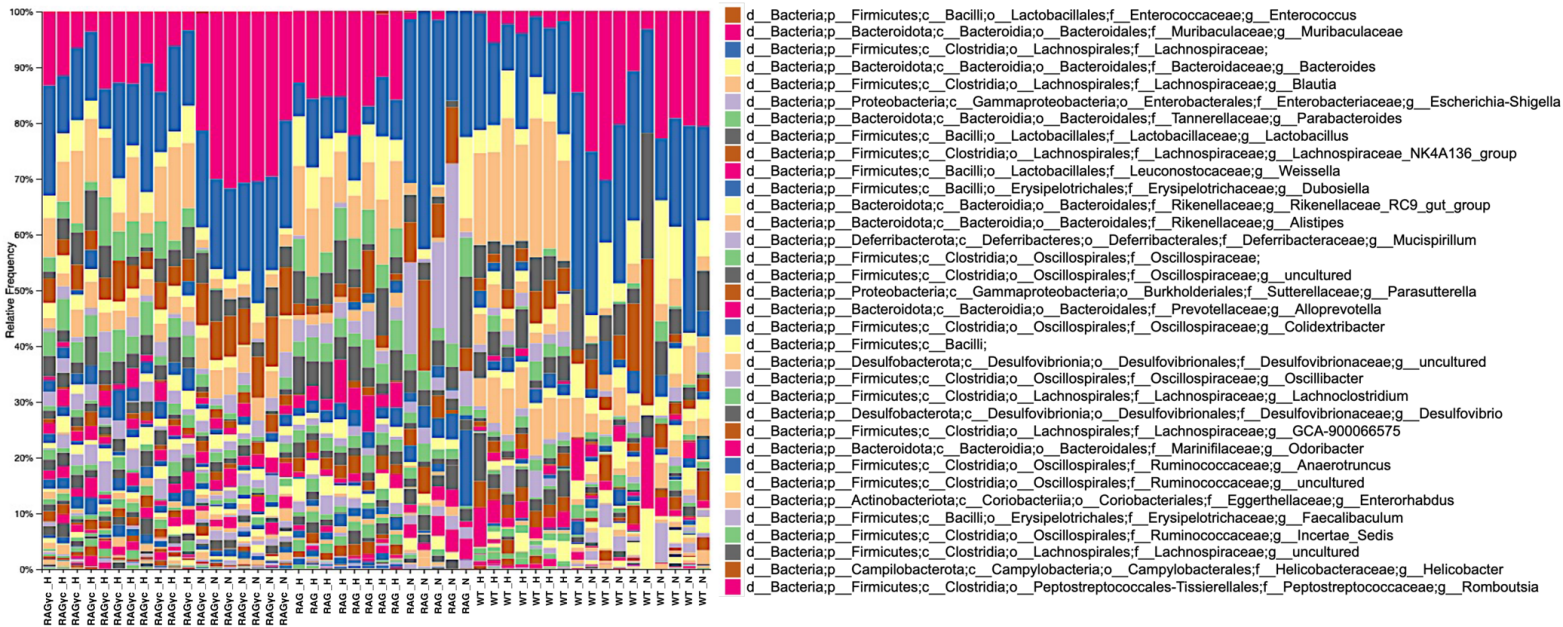
Gut microbiota composition across mouse genotypes. Stacked bar plots display the relative abundance of bacterial genera in faecal samples across the three mouse genotypes (WT, RAG, and RAG*γ*c) and two dietary conditions (normal diet [ND] and high‐fat diet [HFD]). Taxonomic assignments are shown at the genus level, with species‐level annotations provided where available. Each bar represents an individual mouse, and colours correspond to distinct bacterial taxa, as detailed in the accompanying legend. Samples are ordered along the x‐axis according to genotype–diet group (e.g., WT_N = wild‐type on ND; RAG_H = RAG‐deficient on HFD; RAG*γ*c_H = RAG*γ*c‐deficient on HFD), with individual mouse IDs displayed below. Because genus‐level microbiota profiles encompass a large number of taxa, this figure is intended as a *broad visual overview* of community composition rather than a detailed comparative analysis. Several prominent genera, including *Lactobacillus*, *Bacteroides*, *Blautia*, *Alistipes*, and *Escherichia–Shigella*, are readily observed across groups. Clear shifts in dominant taxa are apparent between genotypes and diets, illustrating the combined influence of immune competence and dietary environment on gut microbial community structure.

Figures 2 and 3 establish key host phenotypes in response to *Trichuris muris* infection, namely parasite burden and systemic humoral immunity across mouse genotypes and dietary exposures. While these outcomes highlight immunological differences in worm clearance and antibody production, they do not reveal how such host‐intrinsic and diet influence the gut microbiota itself, which may be both a modulator and a target of helminth‐driven immunomodulation. To address this, we next present the overall taxonomic composition of the gut microbiota across individual mice (Figure 4). This stacked taxonomy bar plot provides a detailed summary of the microbial community structure at the genus level, enabling visual comparison of dominant and subdominant taxa across genotypes.

WT mice fed on a ND, relative to those on HFD, displayed higher relative abundances of taxa such as *Muribaculaceae, Dubosiella*, and *Lactobacillus*. Immune‐deficient genotypes (RAG*γ*c‐KO and RAG‐KO) on ND compared to their counterparts on HFD exhibited increased representation of genera such as *Escherichia–Shigella*, suggestive of dysbiosis. Dietary exposure further modulated microbial profiles: HFD was generally associated with expansion of *Lactobacillaceae*, *Blautia*, and *Alistipes*, *Bacteriodes* across genotypes. Notably, several taxa such as *Faecalibaculum*, *Parabacteroides*, and *Romboutsia* varied in abundance in a genotype‐specific manner, highlighting the interactive effects of host immunity and diet in shaping gut microbial communities. Gut microbiota composition stratified by diet alone is shown in Figure S1.

### 3.4 Gut Microbiota Composition Is Shaped by Diet and Immune Competence More Than Infection Status

Having established distinct taxonomic signatures across mouse genotypes and diets (Figure 4), we next sought to understand how these compositional differences relate to broader microbial community structure. To this end, we profiled overall microbiota composition using 16S rRNA gene sequencing and visualised community‐level differences using principal coordinate analysis (PCoA) based on Bray–Curtis dissimilarity. This approach enabled us to assess how gut microbial diversity clusters across immune genotypes, diets, and infection states.

**FIGURE 5:**
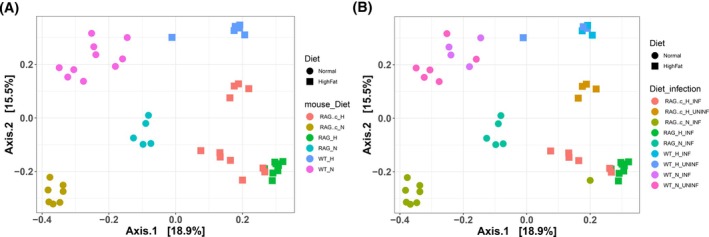
Principal coordinate analysis (PCoA) of gut microbiota profiles stratified by mouse genotype, diet, and infection status. (A) PCoA based on Bray–Curtis dissimilarity shows distinct clustering of microbial communities driven by mouse genotype and dietary exposure. WT_N = wild‐type mice on ND; WT_H = WT mice on HFD; RAG_N = RAG1‐deficient mice on ND; RAG_H = RAG1‐deficient on HFD; RAG*γ*c_N = RAG1/*γ*c‐deficient mice on ND; RAG*γ*c_H = RAG1/*γ*c‐deficient mice on HFD. Colours represent mouse genotype categories, while point shapes denote diet type (ND = circles; HFD = squares). (B) PCoA plot further stratified by infection status. Clustering remained largely defined by genotype and diet, with limited separation between infected and uninfected mice within each group. Colours represent combined mouse genotype–infection categories, while point shapes denote diet type (ND = circles; HFD = squares). PERMANOVA output: Mouse genotype and diet explained a significant proportion of microbiota variance (*R* ^2^ = 0.38595, *p* = 0.001), highlighting the dominant role of these factors in shaping gut microbial composition.

WT mice on a ND (WT_N) formed a tightly clustered group, indicating a stable and consistent microbial community under immunocompetent conditions and conventional feeding. In contrast, WT mice on a HFD (WT_H) clustered separately, demonstrating that diet alone induces substantial shifts in microbiota composition. Similar separation was observed between RAG knockout (RAG_N vs. RAG_H) and RAG1/*γ*c‐deficient (RAG*γ*c_N vs. RAG*γ*c_H) groups, further underscoring the impact of dietary exposure across different immune backgrounds (Figure 5A).

When stratified by infection status (Figure 5B), microbial profiles remained predominantly clustered by genotype and diet, with minimal separation between infected and uninfected mice within each dietary group. This suggests that while *T. muris* infection may influence the microbiome at a more specific taxonomic or functional level, its impact on overall microbial community structure is modest compared to the strong effects of immune status and dietary composition.

PERMANOVA analysis confirmed that the combination of diet and immune competence (mouse_Diet) significantly explained variation in gut microbiota profiles (*R* ^2^ = 38.6%, *p* = 0.001). These results collectively indicate that immune competence and dietary exposure are the dominant factors shaping gut microbial communities in this model, while *T. muris* infection exerts subtler or more localised effects on the microbiome.

Gut microbiota profiles, assessed by principal coordinate analysis (PCoA) using Bray–Curtis dissimilarity, revealed that microbial community structure clustered distinctly by mouse genotype and dietary exposure. WT, RAG1/*γ*c‐deficient, and RAG‐deficient mice each formed separate clusters, with diet further stratifying microbiota profiles within each genotype. PERMANOVA analysis confirmed significant associations between microbiota composition, immune genotype, and diet (*R* ^2^ = 0.39, *p* = 0.001).

Because genotype and diet exerted such strong effects on both microbiota composition and worm burden, we interpret these associations as primarily reflective of the host immune and dietary environment rather than implying a causal microbiota‐driven effect on infection outcome.

### 3.5 Validation of 16S Data Using Shotgun Metagenomics

**FIGURE 6:**
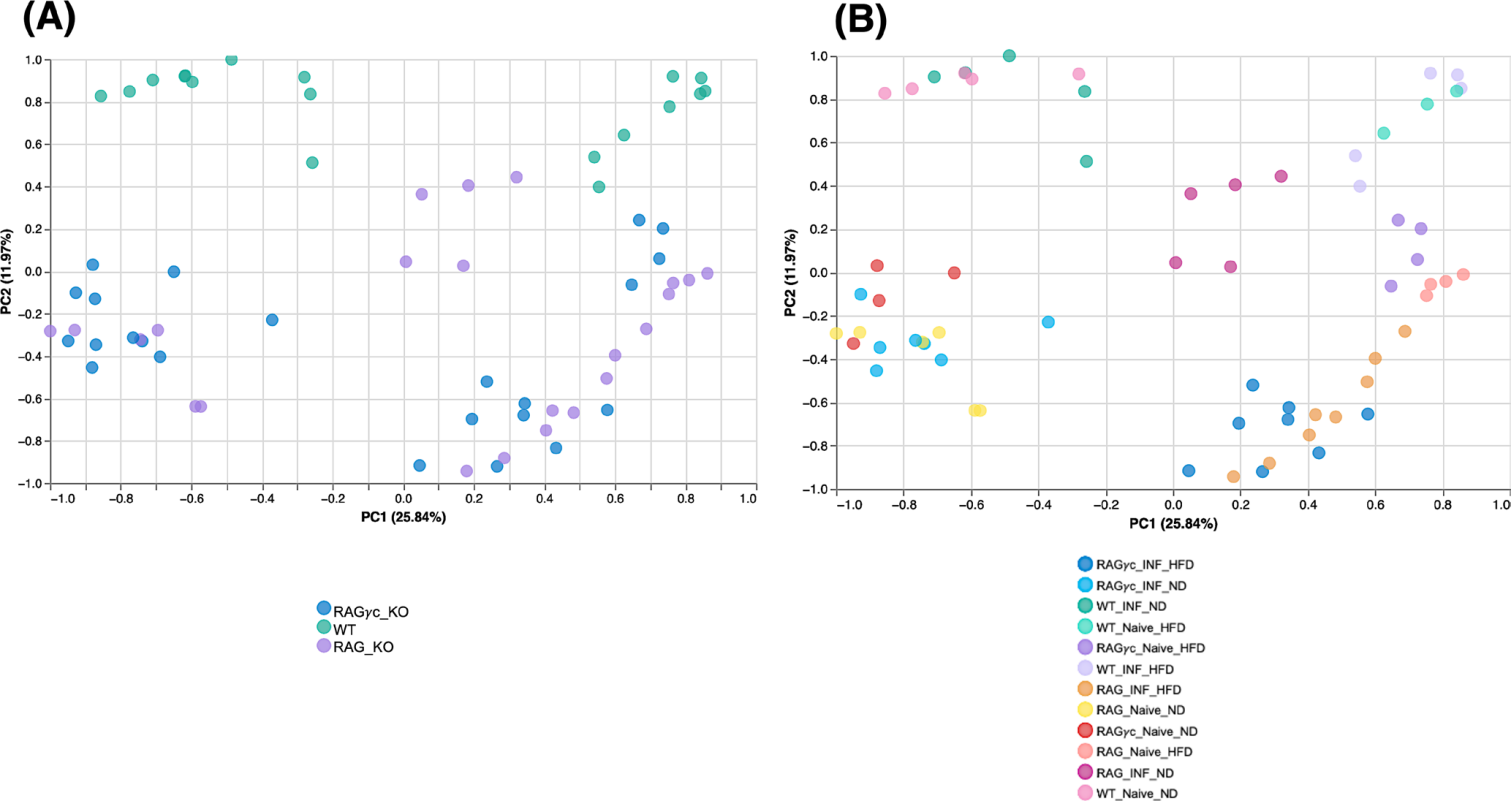
Principal coordinate analysis (PCoA) of gut microbiota profiles based on shotgun metagenomic sequencing. (A) Samples coloured by mouse genotype (RAG*γ*c_KO, WT, RAG_KO) demonstrate distinct clustering driven by immune competence, indicating that host immune architecture is a primary determinant of microbiota structure. WT samples form two sub‐clusters (upper left and upper right). Panel B reveals that this split corresponds largely to diet, indicating that while immune status is a major determinant of microbiota structure, dietary differences within WT mice also create distinct community profiles. RAG mice (lacking adaptive immunity) cluster separately, consistent with a unique microbial community structure shaped by their immunodeficiency. RAG1/*γ*c‐deficient (further impaired immune function) also form their own distinct cluster, showing that the degree of immune impairment strongly influences gut microbial composition. (B) Samples stratified by diet and infection status. Dietary exposure remains a key driver of microbial community clustering, with minimal separation by infection status. This suggests that *T. muris* infection exerts modest influence on global microbiota structure relative to diet and immune status. Mice fed on HFD consistently cluster away from those on ND, across all immune backgrounds. The infection status does not strongly separate samples within the same diet‐immunity groups, mirroring our earlier findings from 16S rRNA analysis. Infection may induce more subtle, taxon‐specific changes rather than global community shifts. Naive mice (uninfected) on HFD sometimes overlap with their infected counterparts.

To extend these findings and achieve greater taxonomic resolution, we performed shotgun metagenomic sequencing on caecal samples. This enabled deeper characterisation of microbial shifts observed in 16S analysis and confirmed the dominant roles of host genotype and diet in shaping the microbiome. In the principal coordinate analysis (PCoA) plot shown in Figure 6A, where samples are stratified by immune genotype (wild‐type, RAG1‐deficient, and RAG1/*γ*c‐deficient mice), gut microbiota profiles clustered according to immune competence. Wild‐type (WT) mice formed a relatively tight cluster, indicating a stable and consistent gut microbiota structure under intact immune conditions. In contrast, RAG1‐deficient, which lack adaptive immunity, clustered separately, reflecting a unique microbial community shaped by their immunodeficiency. RAG1/*γ*c‐deficient mice, with further impaired immune function, also formed their own distinct cluster, demonstrating that increasing degrees of immune impairment strongly influence gut microbiota composition. These observations confirm that host immune architecture is a major determinant of microbiome structure and a contribution of both adaptive and innate lymphoid cells, even when using high‐resolution shotgun metagenomic profiling.

In Figure 6B, where samples are stratified by both dietary exposure and infection status, diet emerged as the dominant factor shaping microbial community structure. Mice fed HFD consistently clustered away from those on ND across all immune genotypes, indicating that dietary composition has a strong and consistent impact on the gut microbiota. Infection status did not significantly separate samples within the same diet‐genotype groups, supporting earlier findings from 16S rRNA analysis that *T. muris* infection induces more subtle, taxon‐specific microbiome changes rather than large‐scale community shifts. Interestingly, naive (uninfected) mice on HFD often overlapped with their infected counterparts, further suggesting that diet exerts a stronger influence on microbiome structure than infection in this experimental context.

### 3.6 Differential Impact of High‐Fat Diet on Gut Microbiota Across Immune Genotypes

To clarify how diet alone alters microbial ecology across different immune backgrounds, we performed differential abundance analysis to compare the gut microbiota profiles of mice fed on a HFD versus a ND within each immune genotype. This stratified approach allowed us to isolate the specific influence of diet within distinct immune backgrounds.

#### 3.6.1 Wild‐Type Mice (WT)

**FIGURE 7:**
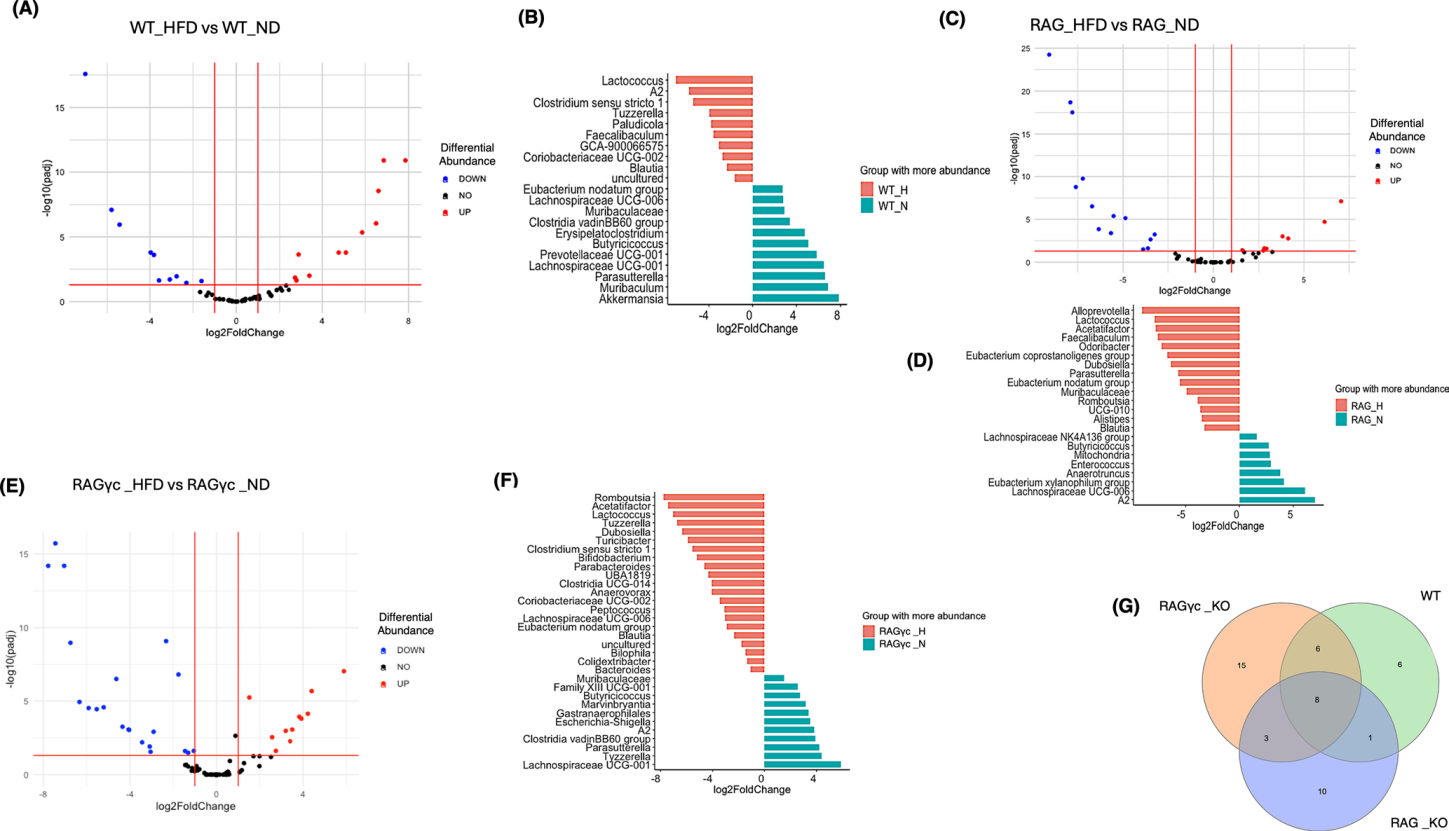
(A) Volcano plot showing differentially abundant taxa in WT mice comparing HFD to ND. Red points indicate taxa significantly enriched in HFD‐fed WT mice (log_2_ fold change > 1, adjusted *p* < 0.05), blue points represent taxa significantly enriched in ND‐fed WT mice, and black points represent non‐significant taxa. (B) Corresponding waterfall plot for WT mice highlighting the key taxa that were significantly enriched in each diet group. WT mice on HFD exhibited increased abundance of *Lactococcus*, *Clostridium sensu* stricto 1, and Tuzzerella, whereas ND‐fed WT mice had higher abundance of *Akkermansia, Muribaculum*, and *Parasutterella*. (C) Volcano plot showing differentially abundant taxa in RAG‐deficient (RAG‐KO) mice comparing HFD to ND groups. Similar to panel A, red and blue points indicate taxa significantly enriched in HFD and ND groups, respectively. (D) Corresponding waterfall plot for RAG‐KO mice. HFD‐fed RAG‐KO mice exhibited enrichment of *Alloprevotella, Lactococcus*, and *Acetatifactor*, while ND‐fed RAG mice had increased abundance of *Lachnospiraceae* NK4A136 group, *Eubacterium xylanophilum* group, and *Anaerotruncus*. (E) Volcano plot displaying differentially abundant taxa in RAG1/*γ*c‐deficient mice comparing HFD to ND groups. Significant taxa enriched in HFD‐fed RAG*γ*c‐KO mice are shown in red, and those enriched in ND‐fed RAG*γ*c‐KO mice are shown in blue. (F) Corresponding waterfall plot for RAG*γ*c mice. HFD‐fed RAG*γ*c‐KO mice showed enrichment of *Romboutsia, Acetatifactor*, *Lactococcus*, and *Tuzzerella*, while ND‐fed RAG*γ*c‐KO mice were enriched in *Lachnospiraceae UCG‐001*, *Tyzzerella*, *Escherichia‐Shigella*, and *Gastranaerophilales*. (G) Venn diagram summarising the overlap of differentially abundant taxa across the three genotypes (WT, RAG‐KO, and RAG*γ*c‐KO). A total of eight microbial taxa were commonly affected by HFD across all mouse types, whereas each genotype also exhibited a substantial number of unique taxa responsive to dietary intervention (21 in WT, 22 in RAG‐KO, and 32 in RAG*γ*c‐KO).

In WT mice, the volcano plot (Figure 7A) revealed a substantial number of significantly differentially abundant taxa between the HFD (10) and ND (11) groups. Key taxa such as *Akkermansia*, *Muribaculum*, and *Parasutterella* were enriched in the ND group, while *Lactococcus* and *Clostridium sensu stricto 1* were significantly enriched in the HFD group (Figure 7B), indicating that HFD reshaped the gut microbial landscape in immunocompetent hosts.

#### 3.6.2 RAG‐Deficient Mice (RAG)

In RAG‐deficient mice dietary impact was also evident as seen in Figure 7C where HFD‐fed mice had 14 enriched taxa compared to 8 taxa that were more enriched in the ND‐fed mice. HFD‐fed RAG‐KO mice exhibited enrichment of taxa such as *Alloprevotella*, *Lactococcus*, *Blautia*, and *Eubacterium coprostanoligenes* group, while *Lachnospiraceae NK4A136* and *Eubacterium xylanophilum* were more abundant in the ND group (Figure 7D). The pronounced microbial shifts in this group suggest that diet can still modulate microbial communities even when key immune components are absent.

#### 3.6.3 RAG1/*γ*c‐Deficient Mice (RAG*γ*c‐KO)

Figure 7E displays the number and statistical significance of differentially abundant bacterial genera between RAG1/*γ*c‐deficient mice fed a HFD (21) versus a ND (11). Genera with significant differences in abundance are highlighted based on adjusted *p*‐value and fold change. The accompanying waterfall plot Figure 7F illustrates the direction and magnitude of change for selected genera, with enrichment of *Romboutsia*, *Acetatifactor*, *Bacteriodes*, and *Lactococcus* in HFD‐fed mice, and higher levels of *Escherichia–Shigella*, *Clostridia vadinBB60*, and *Parasutterella* in mice on a ND.

#### 3.6.4 Shared and Unique Microbial Signatures Across Genotypes

Figure 7G summarising differentially abundant taxa across genotypes revealed that only eight microbial taxa were consistently affected by diet across all mouse types. In contrast, a substantial number of taxa were unique to each genotype, with 21 taxa specific to WT mice, 32 unique to RAG/*γ*c‐deficient mice, and 22 unique to RAG‐KO mice. These findings indicate that while some dietary effects on the microbiome are conserved across immune backgrounds, the majority of microbial responses to diet are strongly genotype‐specific. In WT mice, HFD feeding was associated with an enrichment of *Lactococcus* and *Clostridium sensu stricto 1*, while *Akkermansia*, *Muribaculum*, and *Parasutterella* were more abundant in those on a ND. In RAG/*γ*c‐deficient mice, *Romboutsia*, *Acetatifactor*, and *Lactococcus* were enriched under HFD conditions, whereas *Escherichia‐Shigella* and *Clostridia vadinBB60* dominated in the ND group. Similarly, in RAG‐KO mice, *Alloprevotella*, *Lactococcus*, and *Eubacterium coprostanoligenes* group were enriched under HFD, while *Lachnospiraceae NK4A136* and *Eubacterium xylanophilum* were more abundant in mice fed a ND.

### 3.7 Gut Microbiota Profiles Cluster by Host Immune Background and Diet

We next examined how infection interacts with diet and immune status to modulate microbial community structure. This analysis enabled us to determine whether infection amplifies or overrides diet‐induced differences. We compared microbiota profiles between WT and RAG1/*γ*c‐deficient (RAG*γ*c‐KO) mice following *T. muris* infection under both ND and HFD conditions.

**FIGURE 8:**
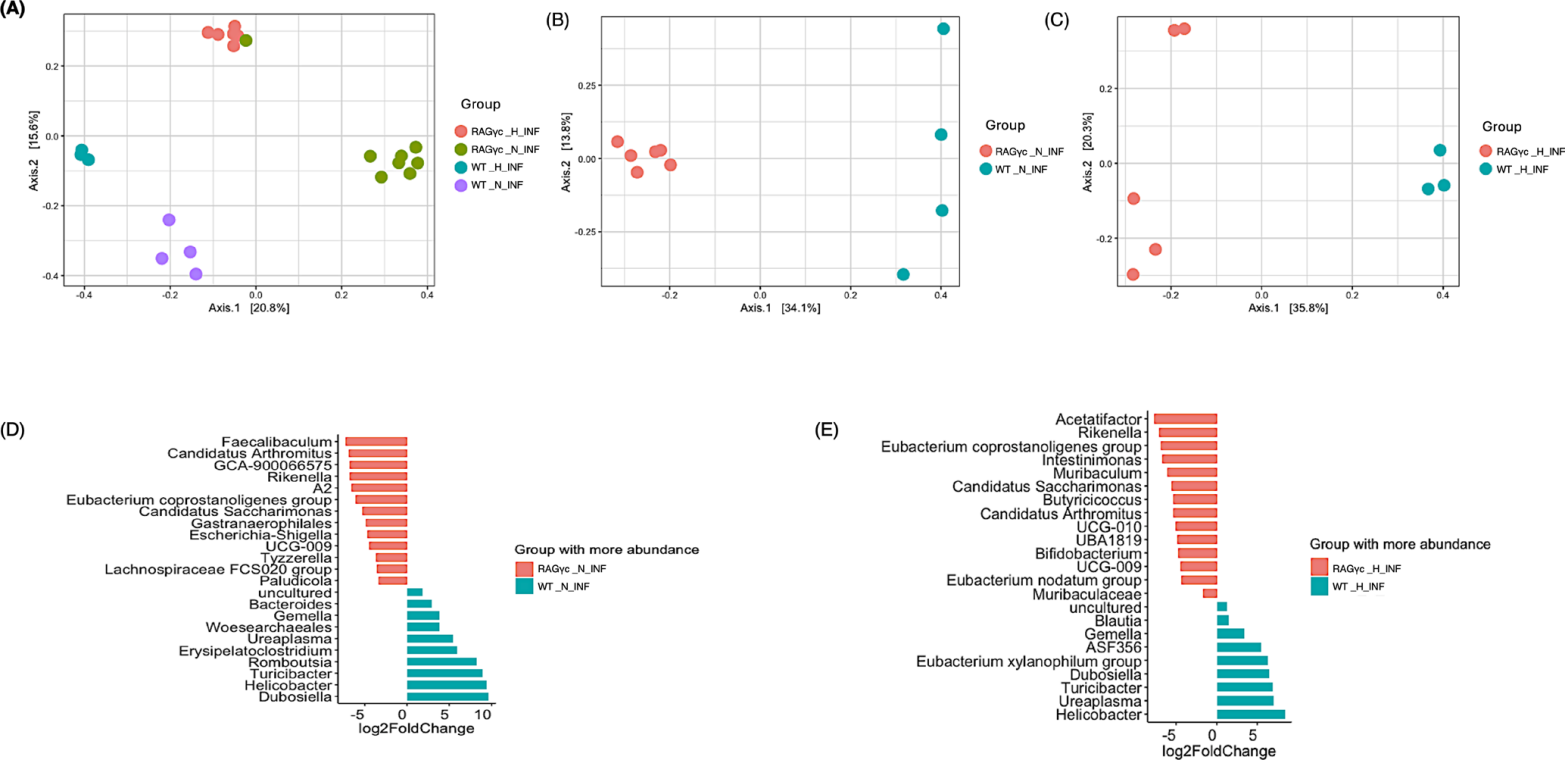
Impact of immune genotype and diet on gut microbiota composition in *T. muris*‐infected mice. (A) Principal coordinate analysis (PCoA) plots based on Bray–Curtis dissimilarity showing gut microbial community structures across wild‐type (WT) and RAG1/*γ*c‐deficient (RAG*γ*c‐KO) mice under ND and HFD conditions. Distinct clustering patterns were observed, with greater separation between genotypes under HFD exposure. These three plots compare microbial community structures between: RAG*γ*c_N_INF versus WT_N_INF (ND) versus WT_H_INF (HFD). PCoA plots B and C show clear separation between RAG1/*γ*c‐deficient and wild‐type mice in ND and HFD groups respectively, suggesting distinct microbial community structures driven by immune genotype. Separation is more pronounced on the HFD, indicating that diet amplifies microbiome differences between these immune backgrounds. (D) Differentially abundant taxa between RAG*γ*c_N_INF and WT_N_INF mice fed a ND. The waterfall plot shows taxa enriched in RAG*γ*c_N_INF (red) and WT_N_INF (blue), with log_2_ fold‐change on the x‐axis. WT_N_INF microbiomes are enriched in beneficial genera like Bacteroides, Turicibacter, and Romboutsia. RAG*γ*c_N_INF microbiomes are enriched in taxa such as Faecalibaculum, Candidatus Arthromitus, Escherichia‐Shigella, and Gastranaerophilales, which may reflect dysbiosis and immune dysfunction. (E) Differentially abundant taxa between RAG*γ*c_H_INF and WT_H_INF mice fed a HFD. Taxa enriched in RAG*γ*c_H_INF are shown in red, and those enriched in WT_H_INF are shown in blue. RAG*γ*c_H_INF mice are enriched in Acetatifactor, Rikenella, and Candidatus Saccharimonas, indicating a distinct microbial profile under HFD. WT_H_INF mice maintain beneficial microbes like Blautia, Turicibacter, Dubosiella, and Helicobacter. RAG*γ*c_N_INF‐infected RAG1/*γ*c‐deficient mice on ND. RAG*γ*c_H_INF‐RAG1/*γ*c‐deficient mice on HFD, WT_N_INF‐infected WT mice on ND, WT_H_INF‐infected Wild type mice on HFD diet.

We observed distinct clustering by both genotype and diet among *T. muris*–infected mice (Figure 8A). RAG1/*γ*c‐deficient and wild‐type mice formed clearly separated clusters, and within each genotype, HFD and ND induced marked shifts in microbial community structure. These findings demonstrate that both immune competence and dietary exposure independently contribute to shaping the gut microbiota, even in the context of helminth infection.

To unravel the specific contribution of host immune status from dietary influence, we next focused on *T. muris*–infected mice maintained on a ND (Figure 8B). Comparing RAG*γ*c‐deficient and wild‐type mice under this controlled dietary condition revealed strong genotype‐dependent clustering. This indicates that the absence of adaptive immune cells together with innate lymphoid cells is sufficient to drive significant alterations in microbial community structure. This focused comparison isolates the immunological effect observed in Figure 8A and establishes immune competence as a primary determinant of microbiota composition.

Finally, to test whether this immune‐driven separation persists under HFD diet, we compared RAG*γ*c‐deficient and wild‐type mice fed a HFD (Figure 8C). Again, PCoA revealed robust genotype‐dependent separation, mirroring the pattern observed under ND. This confirms that loss of the adaptive and innate lymphoid cells consistently reshapes the gut microbiota across dietary contexts. Taken together, these three analyses establish that while both diet and immune status shape the gut microbiome, immune competence exerts a dominant and reproducible influence, regardless of dietary environment.

Under ND, WT mice were enriched for taxa typically associated with gut health, including *Bacteroides*, *Turicibacter*, and *Romboutsia* (Figure 8D). In contrast, RAG*γ*c‐deficient mice exhibited higher abundances of potentially dysbiotic genera such as *Faecalibaculum*, *Candidatus Arthromitus*, *Escherichia–Shigella*, and unclassified members of the *Gastranaerophilales* order, suggesting that the absence of adaptive and innate lymphoid compartments disrupts microbial homeostasis.

Under HFD conditions, genotype‐specific microbial signatures remained evident (Figure 8E). RAG*γ*c‐deficient mice were enriched in taxa such as *Acetatifactor*, *Rikenella*, and *Candidatus Saccharimonas*, whereas WT mice maintained a microbial community dominated by *Blautia*, *Turicibacter*, *Dubosiella*, and *Helicobacter*. These shifts indicate that HFD exacerbates genotype‐driven microbial divergence, potentially amplifying maladaptive microbiota configurations in immunodeficient hosts.

### 3.8 Diet and Infection Independently and Jointly Shape Microbial Diversity in Wild‐Type Mice

To dissect the effects of infection and diet on microbial diversity, we performed principal coordinate analysis (PCoA) of gut microbiota composition across WT mice under varying dietary and infection conditions. We included four distinct PCoA comparisons, each capturing a specific intersection of diet (ND vs. HFD) and infection status (*T. muris*–infected vs. uninfected), allowing us to systematically assess the contributions of each factor to microbial community structure.

**FIGURE 9:**
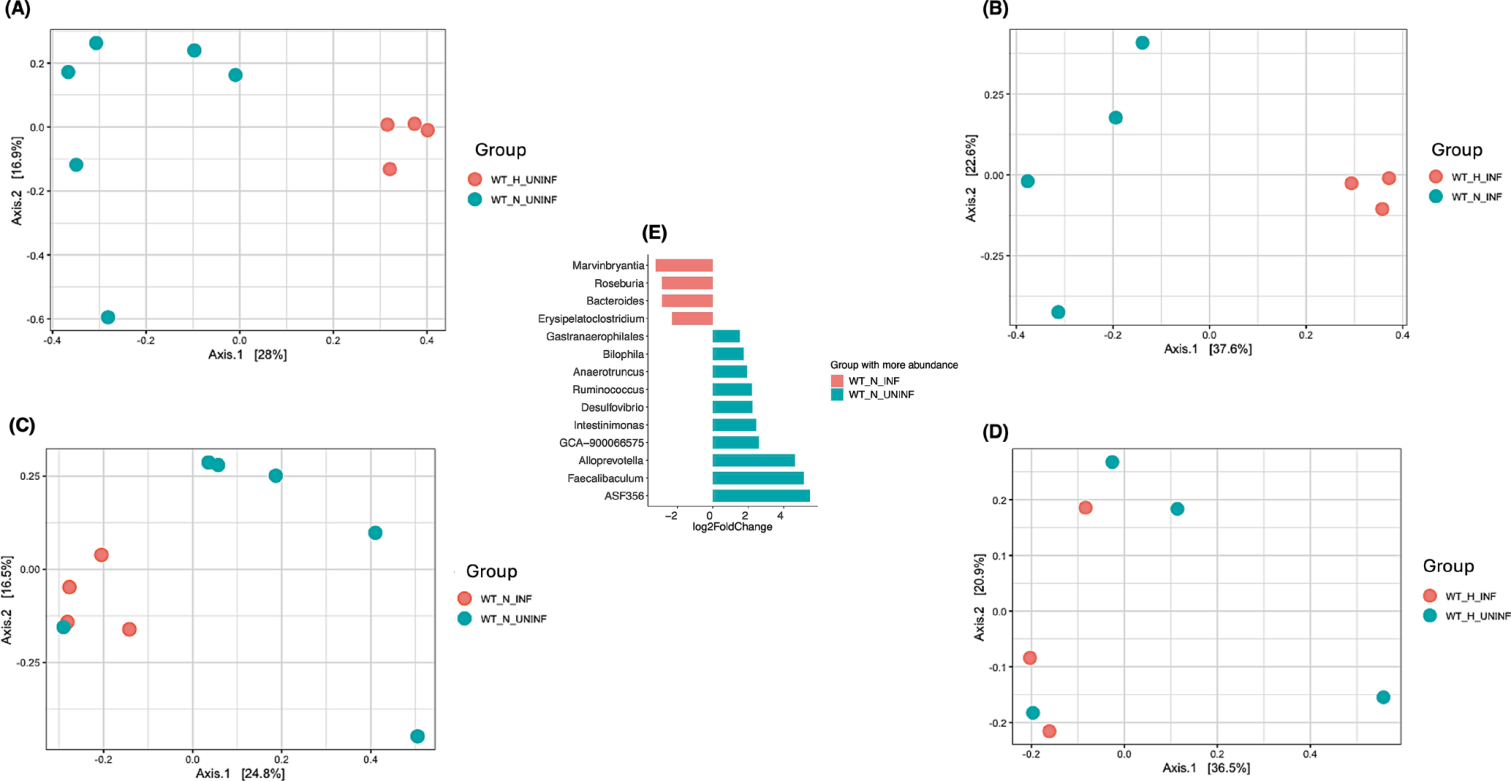
Impact of diet and *T. muris* infection on gut microbiota composition in wild‐type mice. (A) Principal coordinate analysis (PCoA) plot showing microbial clustering of uninfected WT mice on HFD (WT_H_UNINF) versus ND (WT_N_UNINF). (B) PCoA plot comparing infected WT mice on HFD (WT_H_INF) versus ND (WT_N_INF). (C) PCoA plot showing microbial separation between infected and uninfected WT mice on ND. (D) PCoA plot of infected and uninfected WT mice on HFD. (E) Differential abundance analysis comparing WT_N_INF and WT_N_UNINF mice. Taxa enriched in infected mice are shown in red, while those enriched in uninfected mice are shown in blue, with log_2_ fold‐change on the x‐axis.

Under uninfected conditions, comparison of WT mice on ND versus HFD revealed clear separation in microbiota profiles (Figure 9A). Axis 1 and axis 2 accounted for 28.0% and 16.9% of the variance, respectively, indicating that dietary composition alone is sufficient to drive substantial alterations in the gut microbiota, even in the absence of infection.

In the presence of infection, microbial divergence between dietary groups became more pronounced. Infected WT mice fed a HFD clustered distinctly from those on a ND (Figure 9B), with axis 1 and axis 2 explaining 37.6% and 22.6% of the variance, respectively. These results suggest that infection interacts with dietary background to induce greater microbiome restructuring.

Among mice maintained on a ND, infected and uninfected WT mice showed moderate separation in community composition (Figure 9C; axis 1: 24.8%, axis 2: 16.5%), indicating that infection alters the microbiota, but within a relatively stable nutritional environment. In contrast, the HFD amplified these infection‐associated shifts. WT mice on HFD showed a trend toward separation between infected and uninfected groups (Figure 9D), with axis 1 and axis 2 capturing 36.5% and 20.9% of the variance, respectively, consistent with a combined effect of diet and infection, although the clustering was less distinct than under ND conditions.

To identify specific microbial taxa altered by *T. muris* infection, we performed differential abundance analysis comparing infected (WT_N_INF) and uninfected (WT_N_UNINF) wild‐type mice maintained on a ND (Figure 9E). The analysis revealed significant compositional shifts, with infection associated with both enrichment and depletion of distinct bacterial genera.

Infected mice exhibited increased relative abundances of genera such as *Marvinbryantia*, *Roseburia*, *Bacteroides*, and *Erysipelatoclostridium*, suggesting a restructuring of the gut ecosystem in response to helminth exposure. Conversely, uninfected mice harboured higher levels of taxa including *Gastranaerophilales, Bilophila, Anaerotruncus, Ruminococcus, Desulfovibrio, Intestinimonas*, and *Alloprevotella*.

These findings indicate that even in the absence of dietary perturbation, helminth infection drives targeted microbial remodelling, potentially through immune‐mediated or niche‐altering mechanisms. When interpreted alongside diversity and clustering analyses (Figure 9A–D), these taxon‐level changes provide insight into how infection selectively modulates gut microbial communities beyond the overall shifts in composition.

In contrast to the infection‐driven microbial shifts observed under normal dietary conditions, differential abundance analysis revealed no significantly altered genera between infected and uninfected WT mice fed a HFD. Given the magnitude of the diet‐associated shifts observed in overall community structure, any infection‐related effects appeared comparatively small and did not meet our detection thresholds in differential abundance testing. We observed consistent reductions in microbial alpha diversity associated with HFD, immune deficiency, and *T. muris* infection. These effects were evident across gut compartments and sequencing platforms, including 16S rRNA and shotgun metagenomics (Figures S2–S4). Genotype‐, diet‐, and infection‐specific comparisons further revealed that immune‐compromised mice, particularly under dietary and infectious stress, exhibited the greatest losses in alpha microbial diversity.

### 3.9 Diet and Infection Influence Microbial Functional Potential

**FIGURE 10:**
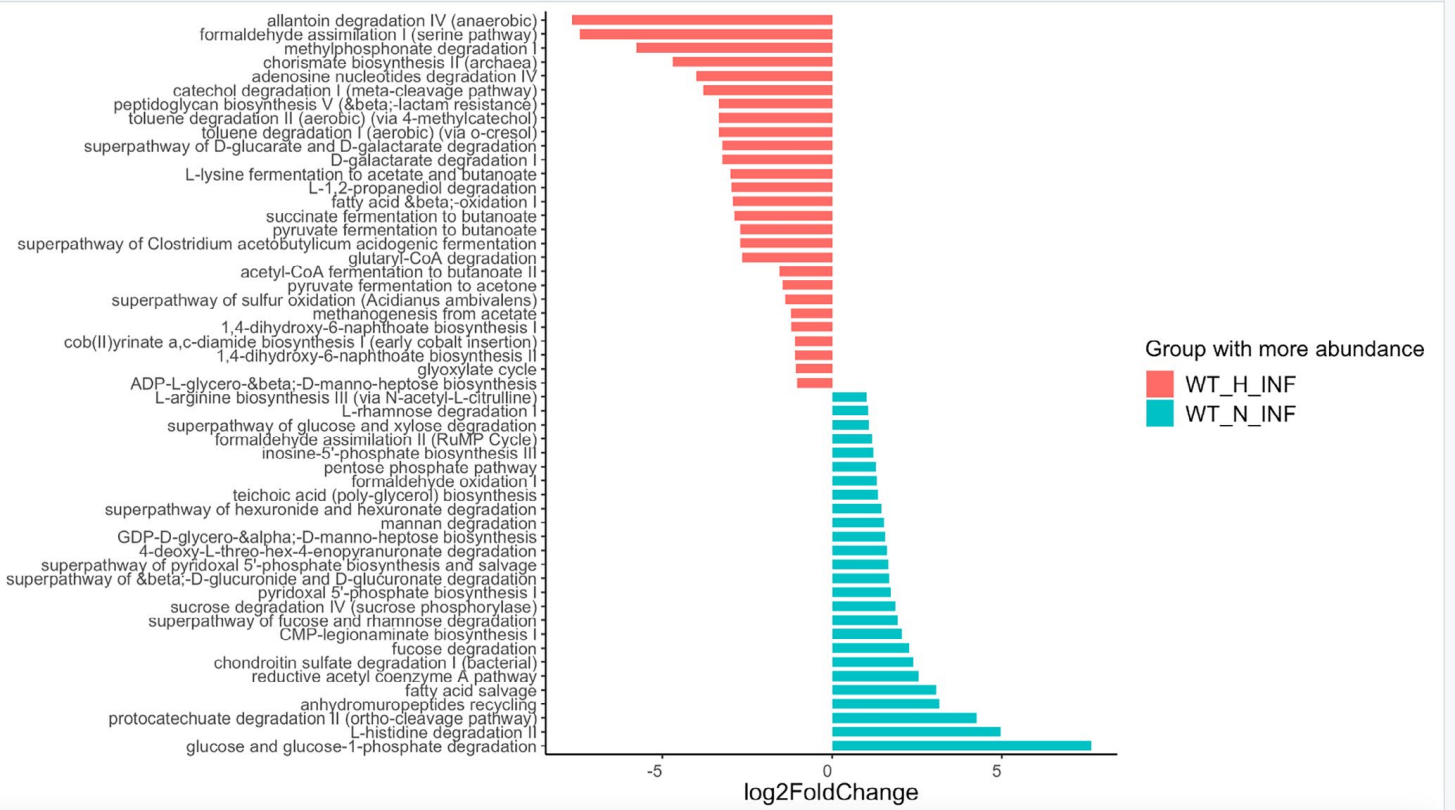
Differentially abundant microbial metabolic pathways in infected wild‐type mice fed a HFD versus ND. Functional prediction analysis using PICRUSt2 identified pathways significantly enriched in the microbiomes of infected wild‐type mice on HFD (WT_H_INF, red) and those on ND (WT_N_INF, blue). The x‐axis represents log_2_ fold change in pathway abundance. WT_H_INF mice exhibited enrichment of pathways related to amino acid degradation, aromatic compound degradation, and fatty acid metabolism. In contrast, WT_N_INF mice showed enrichment of carbohydrate and nucleotide degradation and biosynthesis pathways.

Functional pathway analysis using PICRUSt2 (Figure 10)provided insight into how dietary environment modulates microbiome function during infection. Infected WT mice on a HFD showed significant enrichment of amino acid degradation pathways, butanoate fermentation, fatty acid *β*‐oxidation, and aromatic compound degradation that coincided with their enhanced resistance to *T. muris* infection. Conversely, infected WT mice on a ND, which were more susceptible to *T. muris* infection, exhibited higher abundance of pathways involved in carbohydrate degradation, nucleotide biosynthesis, and glycan metabolism. These shifts suggest that HFD reprograms the microbiome's metabolic capacity in infected hosts, potentially impacting host energy balance and immune responses.

These findings suggest that HFD exposure alters the metabolic capacity of the gut microbiome, potentially impacting host energy balance, immune responses, and helminth‐host interactions.

## 4 Discussion

Our study demonstrates that host immune competence and diet interact to shape the outcomes of *Trichuris muris* infection and gut microbiota composition. Across mouse models of varying immune capability, we observed that adaptive immunity plays a critical role in parasite expulsion and both innate and adaptive immunity play key roles in the structuring of gut microbial communities, with the diet further modulating these effects.

RAG‐KO mice, which lack adaptive immunity, and RAG*γ*c‐KO mice which lack adaptive and innate lymphoid cells unsurprisingly harboured persistent full worm burdens from a low dose infection, consistent with the essential role of immunity in parasite control [8]. WT mice on a normal diet also failed to expel worms from a low dose infection as reflected by worm recovery at day 35 and establishing chronic infection as previously shown [8]. WT mice on a ND (WT_N) showed robust parasite‐specific antibody responses, particularly IgG2a/c consistent with a Th1‐skewed profile. In the context of *T. muris*, such IgG2a/c‐dominated responses are characteristic of chronic infection [18, 19]. The expulsion of worms in WT mice on a HFD (WT_HFD) was accompanied by elevated IgG1 titres and reduced IgG2a/c levels, suggesting a diet‐induced shift toward a Th2‐skewed immune response. Our findings are consistent with recent work by Funjika et al. [16], who showed that provision of a high‐fat diet enabled WT mice to expel worms, supporting the idea that dietary modulation can unlock protective immune responses and overcome the chronicity otherwise observed in WT animals under normal diet. Their study showed that dietary fat primes CD4^+^ T cells to upregulate the IL‐33 receptor ST2, leading to amplified Th2 cytokine production upon IL‐33 stimulation. Together, these data support the idea that dietary lipids can reshape immune responses toward a Th2‐dominant profile, enhancing protection against *T. muris* infection.

Our observations further suggested that the gut microbiota might contribute to or be modulated by the observed variation in parasite clearance across the different diets and immunocompetence models. Given the established role of the microbiome in shaping immunity [20] and helminth susceptibility [15, 21, 22], as well as the effect of HFD on the gut microbiome [23], we hypothesized that differences in microbial composition could impact the divergent resistance phenotypes observed here.

Our findings show that both host immune competence and dietary exposure are important in shaping the gut microbiota, exceeding the impact of *T. muris* infection on microbial community structure. When compared to RAG*γ*c‐KO mice on ND, WT mice on a ND showed consistent immunocompetent‐associated microbiota characterised by high relative abundances of genera such as *Bacteroides* and *Lactobacillus*, while immune‐deficient mice showed signatures of dysbiosis, including enrichment of *Escherichia–Shigella* and *Candidatus Saccharimonas*. HFD further disrupted microbial composition across all genotypes, increasing taxa such as *Blautia* and *Alistipes*. These observations align with previous work showing that host genotype and diet independently and interactively shape gut microbiota composition [24, 25, 26], with immune deficiency often predisposing to dysbiotic shifts [27, 28] and HFD promoting inflammation‐associated taxa [23]. Thus, we refrain from inferring a causal role for microbiota differences in shaping infection phenotypes. Instead, we interpret our findings as identifying correlative microbial signatures that are associated with diet‐ and immunity‐driven changes in host susceptibility. Definitive mechanistic work, such as microbiota transfer, gnotobiotic colonisation, or immune–specific interventions, will be required to establish whether individual microbial taxa actively contribute to parasite resistance.

In support of our findings, recent work by Le and colleagues demonstrated that dietary cholesterol directly modulates the gut microbiome, selectively enriching microbial taxa such as *Bacteroides* and *Parabacteroides* in both mice and humans [23]. These cholesterol‐interacting microbes also exhibited enriched bile acid–like and sulfotransferase‐like gene functions, underpinning their metabolic adaptation to lipid‐rich environments. Our observation of *Bacteroides* and *Parabacteroides* enrichment in HFD‐fed mice therefore likely shows similar microbial sensing and metabolism of dietary lipids, which may contribute to a gut milieu that modulates host immunity and helminth susceptibility through microbiota–lipid–host interactions.

The enhanced resistance to *T. muris* observed in HFD–fed mice may be partly driven by diet‐induced shifts in gut microbial composition. Several taxa enriched in these mice, including *Faecalibacterium*, *Blautia*, *Lactococcus*, *Parabacteroides*, and *Bacteroides*, have established roles in regulating mucosal immunity [29, 30], promoting epithelial barrier integrity [31], and shaping anti‐inflammatory responses [30, 32, 33]. For instance, *Faecalibacterium prausnitzii* and *Blautia* spp. are prominent butyrate producers, known to reinforce barrier function and promote regulatory T cell differentiation via SCFA‐mediated pathways [29, 30]. Similarly, *Lactococcus lactis* has been linked to enhanced mucosal IgA production and modulation of CD4^+^ T cell responses [30, 31], while *Parabacteroides* spp. have demonstrated the capacity to reduce systemic inflammation and support gut homeostasis in metabolic contexts [33, 34].

These microbial changes may provide a mechanistic link between diet, immune system, and resistance to *T. muris* infection. By promoting a mucosal environment that favours type 2 immunity while maintaining epithelial integrity and suppressing low‐grade inflammation, the HFD‐associated microbiota may inadvertently enhance the host's capacity to expel helminths. The enrichment of *Alistipes* and *Bacteroides*, typically associated with metabolic inflammation [32, 35], may also contribute to controlled immune priming without overt pathology. Thus, rather than uniformly impairing immunity, HFD may alter microbial communities in a way that enhances specific protective responses, thereby highlighting the nuanced role of the microbiome in diet–immune–parasite interactions.

Notably, *T. muris* infection did not produce global shifts in microbiota structure, suggesting that *T. muris* infection effects may be taxon‐specific as reported in earlier research [36, 37]. This limited community‐wide restructuring could reflect the immunomodulatory strategies of chronic *T. muris* infection, which often stabilise host–microbe interactions rather than provoke widespread dysbiosis. Alternatively, infection‐induced changes may be more pronounced at the functional gene or metabolite level—features not captured by 16S‐based taxonomy. Future metagenomic or metabolomic profiling could help resolve whether helminth–microbiome interactions unfold through targeted alterations in metabolic pathways or microbe–immune links.

Immune‐deficient RAG knockout (RAG) and RAG/*γ*c‐deficient mice, as expected, did not expel their worms irrespective of dietary exposure, though HFD further influenced microbial shifts in these groups. As such, even in the absence of adaptive and innate immunity, the microbiome remained responsive to dietary changes, reinforcing the potent influence of diet in shaping microbial ecosystems independent of immune surveillance.

Given the pronounced resistance to *T. muris* infection observed in HFD–fed wild‐type mice, we examined how infection status influenced gut microbial composition within this group compared to their ND counterparts. In WT mice on a ND, several taxa were differentially abundant between infected and uninfected mice. Specifically, microbes such as *Marvinbryantia*, *Roseburia*, *Bilophila*, and *Romboutsia* that have been previously associated with mucosal immune responses [38, 39, 40, 41], bile acid metabolism [42, 43], and inflammation [38], were more abundant in infected mice, suggesting that they either respond to or contribute to the immune responses during *T. muris* infection. Conversely, *Bilophila*, *Anaerotruncus*, and *Desulfovibrio* were enriched in uninfected mice and have been implicated in maintaining mucosal barrier integrity and modulating host immunity [44, 45, 46, 47, 48, 49], suggesting a potential protective or immunoregulatory role in the absence of infection. Their depletion in infected mice may reflect a shift toward a more inflammation‐prone or permissive microbial state that facilitates colonisation by *T. muris* infection. When we conducted the same analysis in WT mice fed a HFD, no differentially abundant taxa were detected between infected and uninfected groups. This lack of microbial changes may reflect a pre‐existing HFD‐driven microbial structure that resists further reshaping upon infection. This may underpin the enhanced resistance to infection observed in this group, potentially through pre‐activation of the innate immunity or altered metabolite production.

Comparing infected WT mice on a HFD versus those on a ND was critical to uncover how diet shapes microbial functional capacity during infection. The enrichment of amino acid degradation, butanoate fermentation, fatty acid *β*‐oxidation, and aromatic compound degradation pathways in infected mice fed on HFD suggests a microbiome adapted for energy‐dense substrate utilisation and anti‐inflammatory metabolite production [50, 51, 52, 53]. Butanoate, for instance, is known to support epithelial barrier integrity and modulate immune responses, potentially reinforcing Th2 polarisation [54, 55, 56]. These functional changes align with the enrichment of butyrate‐producing taxa such as *Faecalibacterium* and *Blautia*, as well as *Parabacteroides* and *Lactococcus*, which have established roles in epithelial protection and immune modulation. In contrast, infected WT mice on a ND exhibited greater enrichment of carbohydrate degradation, nucleotide biosynthesis, and glycan metabolism pathways, indicative of a microbiome oriented toward rapid bacterial growth and mucosal carbohydrate processing [57]. Such a profile may be less conducive to the immunoregulatory or protective effects observed under HFD, potentially contributing to the increased susceptibility observed in these animals.

One limitation of our study is that worm burden was quantified at a single terminal time point. Therefore, while HFD‐fed mice exhibited markedly reduced parasite loads across all genotypes, we cannot fully distinguish whether this reflects enhanced clearance or impaired early establishment of *T. muris*. Much as previous work from our group suggests that HFD can augment type‐2 immune pathways that promote accelerated expulsion [16], longitudinal sampling would be necessary to resolve these kinetics. Future experiments incorporating multiple stages of infection will be important for dissecting these temporal dynamics.

Complementary studies in helminth‐endemic human populations are essential to determine whether similar diet–microbiota–immunity relationships are conserved in natural settings, and to identify population‐specific microbial signatures or dietary patterns associated with susceptibility or resilience. Ultimately, these insights may inform the development of microbiome‐targeted or dietary interventions, such as prebiotics, probiotics, or precision microbiota engineering to bolster host resistance against intestinal helminths.

## 5 Conclusion

Our findings reveal a complex interplay between host immunity, diet, and the gut microbiota in determining resistance to *Trichuris muris* infection. While adaptive immunity is essential for parasite expulsion, a HFD may enhance protective responses by shifting the microbiota toward taxa and functional pathways associated with epithelial integrity and immune modulation. The enrichment of butanoate fermentation and fatty acid *β*‐oxidation pathways, alongside increases in butyrate‐producing and immunoregulatory taxa, suggests that microbial metabolism may actively reinforce type 2 immunity and barrier function in the gut. Even in the absence of adaptive immunity, diet retained a strong influence over microbial community structure and function, underpinning the importance of diet in shaping the gut microbiome profiles.

## Author Contributions

B.W. performed microbiome and immunological data analysis, and wrote the manuscript. B.W., K.S.H., M.A.E.L., S.T., and A.J.B. performed mouse experiments, sample processing, and laboratory assays. A.M.E. and R.K.G. provided overall supervision, conceptual guidance, and critical manuscript revision. All authors contributed to data interpretation, reviewed the manuscript, and approved the final version.

## Funding

BW was partially supported by GCRF collaborative Grant (R120442) from the Royal Society awarded to Professors Richard Grencis and Alison Elliott; he is also partially funded by the National Institute for Health Research (NIHR) under its Global Health Research Group on Vaccines for Vulnerable People in Africa (VAnguard) (Grant Reference Number: NIHR134531), using UK aid from the UK Government to support global health research. This project has also been supported by Wellcome Trust Investigator Award Z10661/Z/18/Z awarded to Professor Richard Grencis and the Wellcome Centre for Cell Matrix Research Grant 088785/Z/09/Z. The views expressed in this publication are those of the author(s) and not necessarily those of the NIHR or the UK Government. BW is based at the MRC/UVRI and LSHTM Uganda Research Unit which is jointly funded by the UK Medical Research Council (MRC) and the UK Department for International Development (DFID) under the MRC/DFID Concordat agreement.

## Supporting information


**Figure S1:** Gut microbiota composition across diet types. Stacked bar plots representing the relative abundance of bacterial genera across individual faecal samples. Taxa are shown at the genus level, with taxonomy annotated down to species where available. Each bar represents one sample, and colours correspond to distinct bacterial taxa, as indicated in the legend on the right.
**Figure S2:** Comparison of alpha diversity in wild‐type WT mice fed either a ND or a HFD. (A) Shannon diversity index of faecal microbiota based on 16S rRNA gene sequencing, showing significantly reduced microbial diversity in WT mice fed a HFD (WT_H) compared to those on a ND (WT_N) (*p* = 0.0027, Wilcoxon test). (B) Shannon diversity index of caecal microbiota based on shotgun metagenomic sequencing. A trend toward reduced diversity in WT_HFD compared to WT_Normal was observed (*p* = 0.06, Wilcoxon test). These results suggest a consistent diet‐associated reduction in microbial diversity across different gut compartments and sequencing platforms.
**Figure S3:** Gut microbial alpha diversity (Shannon index) in naive and infected mice across immune genotypes and dietary groups. (A–C) Comparisons of Shannon diversity in naive (uninfected) mice. (A) Alpha diversity by immune genotype (WT, RAG‐KO, RAG*γ*c‐KO), regardless of diet. (B) Diversity stratified by both immune genotype and diet (ND vs. HFD). (C) Focused comparison within RAG‐KO naive mice, showing reduced diversity in mice on a HFD (RAG_H) versus ND (RAG_N). (D–F) Corresponding comparisons now including infected mice, to assess how *Trichuris muris* infection modifies gut microbial diversity across the same strata. (D) Diversity by genotype (infected vs. naive mice combined), (E) Stratified by both genotype and diet, (F) Focused comparison within RAG‐KO mice on HFD versus ND under infection.
**Figure S4:** Microbial diversity (Shannon index) across different mouse genotypes under varying dietary and infection conditions. (A) Naive RAG*γ*c‐KO, RAG‐KO, and WT mice on a ND, profiled using 16S rRNA sequencing of faecal samples. (B) Infected RAG*γ*c‐KO, RAG‐KO, and WT mice on a ND, also assessed by 16S rRNA sequencing of faecal samples. (C) Infected RAG*γ*c‐KO, RAG‐KO, and WT mice on a ND, profiled via shotgun metagenomics of faecal samples. (D) Naive RAG*γ*c‐KO, RAG‐KO, and WT mice on a HFD, analysed using 16S rRNA sequencing of faecal samples. (E) Infected RAG*γ*c‐KO, RAG‐KO, and WT mice on a HFD, with microbial diversity assessed from faecal samples using 16S rRNA sequencing. (F) Infected RAG*γ*c‐KO, RAG‐KO, and WT mice on a HFD, profiled by shotgun metagenomics of caecal content. This comparative design highlights genotype‐specific shifts in microbial diversity across dietary and infectious states, using both amplicon‐ and shotgun‐based profiling approaches.
**Table S1:** Pairwise post hoc comparisons of intestinal worm burdens across genotype–diet groups.
Dunn's post hoc tests were performed following a significant Kruskal–Wallis test (*p* = 5.8 × 10^−5^) to assess pairwise differences in worm counts between wild‐type (WT), RAG‐deficient (RAG), and RAG*γ*c‐deficient (RAG*γ*c) mice fed either a normal diet (ND) or high‐fat diet (HFD). The table reports the test statistic, unadjusted *p*‐values, Benjamini–Hochberg–adjusted *p*‐values (*p*.adj), and significance calls. Significant comparisons (*p*.adj < 0.05) highlight both genotype‐dependent effects (e.g., impaired parasite control in RAG mice) and diet‐specific effects within genotypes (e.g., improved clearance in WT mice on HFD).

## Data Availability

The data that support the findings of this study are available from the corresponding author upon reasonable request.

## References

[1] H. Behniafar , M. Sepidarkish , M. J. Tadi , et al., “The Global Prevalence of Trichuris Trichiura Infection in Humans (2010‐2023): A Systematic Review and Meta‐Analysis,” Journal of Infection and Public Health 17, no. 5 (2024): 800–809. 38537575 10.1016/j.jiph.2024.03.005

[2] L. Stephenson , C. Holland , and E. Cooper , “The Public Health Significance of Trichuris Trichiura,” Parasitology 121, no. S1 (2000): S73–S95. 11386693 10.1017/s0031182000006867

[3] M. Peradotto , E. Rolle , T. Zaccaria , et al., “An Unpleasant Souvenir: Endoscopic Finding of Trichuris Trichiura (Nematoda: Trichuridae),” Parasitology International 80 (2021): 102220. 33137503 10.1016/j.parint.2020.102220

[4] K.‐S. Ok , Y.‐S. Kim , J.‐H. Song , et al., “Trichuris Trichiura Infection Diagnosed by Colonoscopy: Case Reports and Review of Literature,” Korean Journal of Parasitology 47, no. 3 (2009): 275–280. 19724702 10.3347/kjp.2009.47.3.275 PMC2735694

[5] B. M. Popkin , L. S. Adair , and S. W. Ng , “Global Nutrition Transition and the Pandemic of Obesity in Developing Countries,” Nutrition Reviews 70, no. 1 (2012): 3–21. 22221213 10.1111/j.1753-4887.2011.00456.x PMC3257829

[6] I. C. Bygbjerg , “Double Burden of Noncommunicable and Infectious Diseases in Developing Countries,” Science 337, no. 6101 (2012): 1499–1501. 22997329 10.1126/science.1223466

[7] I. Kolčić , “Double Burden of Malnutrition: A Silent Driver of Double Burden of Disease in Low–and Middle–Income Countries,” Journal of Global Health 2, no. 2 (2012): 020303. 23289074 10.7189/jogh.02.020303 PMC3529312

[8] S. A. Colombo and R. K. Grencis , “Immunity to Soil‐Transmitted Helminths: Evidence From the Field and Laboratory Models,” Frontiers in Immunology 11 (2020): 1286. 32655568 10.3389/fimmu.2020.01286 PMC7324686

[9] K. Hashimoto , R. Uchikawa , T. Tegoshi , K. Takeda , M. Yamada , and N. Arizono , “Depleted Intestinal Goblet Cells and Severe Pathological Changes in SCID Mice Infected With Heligmosomoides Polygyrus,” Parasite Immunology 31, no. 8 (2009): 457–465. 19646210 10.1111/j.1365-3024.2009.01123.x

[10] K. Koyama , H. Tamauchi , and Y. Ito , “The Role of CD4+ and CD8+ T Cells in Protective Immunity to the Murine Nematode Parasite Trichuris Muris,” Parasite Immunology 17, no. 3 (1995): 161–165. 7792100 10.1111/j.1365-3024.1995.tb01018.x

[11] P. D. Cani , “Dietary Emulsifiers—Sweepers of the Gut Lining?,” Nature Reviews Endocrinology 11, no. 6 (2015): 319–320. 10.1038/nrendo.2015.59 25869573

[12] B. Chassaing , O. Koren , J. K. Goodrich , et al., “Dietary Emulsifiers Impact the Mouse Gut Microbiota Promoting Colitis and Metabolic Syndrome,” Nature 519, no. 7541 (2015): 92–96. 25731162 10.1038/nature14232 PMC4910713

[13] H. Ullah , S. Arbab , Y. Tian , et al., “Crosstalk Between Gut Microbiota and Host Immune System and Its Response to Traumatic Injury,” Frontiers in Immunology 15 (2024): 1413485. 39144142 10.3389/fimmu.2024.1413485 PMC11321976

[14] D. Zheng , T. Liwinski , and E. Elinav , “Interaction Between Microbiota and Immunity in Health and Disease,” Cell Research 30, no. 6 (2020): 492–506. 32433595 10.1038/s41422-020-0332-7 PMC7264227

[15] A. Houlden , K. S. Hayes , A. J. Bancroft , et al., “Chronic Trichuris Muris Infection in C57BL/6 Mice Causes Significant Changes in Host Microbiota and Metabolome: Effects Reversed by Pathogen Clearance,” PLoS One 10, no. 5 (2015): e0125945. 25938477 10.1371/journal.pone.0125945 PMC4418675

[16] E. Funjika , S. A. Colombo , K. S. Hayes , et al., “High‐Fat Diet‐Induced Resistance to Helminth Infection via Alternative Induction of Type 2 Immunity,” Mucosal Immunology 16, no. 1 (2023): 27–38. 36690078 10.1016/j.mucimm.2023.01.004

[17] P. Amplicon , P. Clean‐Up , and P. Index , 16s Metagenomic Sequencing Library Preparation (Illumina, 2013).

[18] K. Koyama and Y. Ito , “Comparative Studies on Immune Responses to Infection in Susceptible B10. BR Mice Infected With Different Strains of the Murine Nematode Parasite Trichuris Muris,” Parasite Immunology 18, no. 5 (1996): 257–263. 9229378 10.1046/j.1365-3024.1996.d01-92.x

[19] J. E. Klementowicz , M. A. Travis , and R. K. Grencis , Trichuris Muris: A Model of Gastrointestinal Parasite Infection. Seminars in Immunopathology (Springer, 2012). 10.1007/s00281-012-0348-2 PMC3496546 23053395

[20] Y. Belkaid and T. W. Hand , “Role of the Microbiota in Immunity and Inflammation,” Cell 157, no. 1 (2014): 121–141. 24679531 10.1016/j.cell.2014.03.011 PMC4056765

[21] P. Loke , S. C. Lee , and O. O. Oyesola , “Effects of Helminths on the Human Immune Response and the Microbiome,” Mucosal Immunology 15, no. 6 (2022): 1224–1233. 35732819 10.1038/s41385-022-00532-9

[22] F. Ling , N. Steinel , J. Weber , et al., “The Gut Microbiota Response to Helminth Infection Depends on Host Sex and Genotype,” ISME Journal 14, no. 5 (2020): 1141–1153. 32005978 10.1038/s41396-020-0589-3 PMC7174316

[23] H. H. Le , M.‐T. Lee , K. R. Besler , J. M. Comrie , and E. L. Johnson , “Characterization of Interactions of Dietary Cholesterol With the Murine and Human Gut Microbiome,” Nature Microbiology 7, no. 9 (2022): 1390–1403. 10.1038/s41564-022-01195-9 PMC9417993 35982311

[24] R. N. Carmody , G. K. Gerber , J. M. Luevano , et al., “Diet Dominates Host Genotype in Shaping the Murine Gut Microbiota,” Cell Host & Microbe 17, no. 1 (2015): 72–84. 25532804 10.1016/j.chom.2014.11.010 PMC4297240

[25] T. Dapa , R. S. Ramiro , M. F. Pedro , I. Gordo , and K. B. Xavier , “Diet Leaves a Genetic Signature in a Keystone Member of the Gut Microbiota,” Cell Host & Microbe 30, no. 2 (2022): 183–199.e10. 35085504 10.1016/j.chom.2022.01.002

[26] L. Benga , A. Rehm , C. Gougoula , et al., “The Host Genotype Actively Shapes Its Microbiome Across Generations in Laboratory Mice,” Microbiome 12, no. 1 (2024): 256. 39639355 10.1186/s40168-024-01954-2 PMC11619136

[27] S. Kawamoto , M. Maruya , L. M. Kato , et al., “Foxp3+ T Cells Regulate Immunoglobulin a Selection and Facilitate Diversification of Bacterial Species Responsible for Immune Homeostasis,” Immunity 41, no. 1 (2014): 152–165. 25017466 10.1016/j.immuni.2014.05.016

[28] M. Zhang , K. Sun , Y. Wu , Y. Yang , P. Tso , and Z. Wu , “Interactions Between Intestinal Microbiota and Host Immune Response in Inflammatory Bowel Disease,” Frontiers in Immunology 8 (2017): 942. 28855901 10.3389/fimmu.2017.00942 PMC5558048

[29] S. M. Holmberg , R. H. Feeney , P. K. V. Prasoodanan , et al., “The Gut Commensal Blautia Maintains Colonic Mucus Function Under Low‐Fiber Consumption Through Secretion of Short‐Chain Fatty Acids,” Nature Communications 15, no. 1 (2024): 3502. 10.1038/s41467-024-47594-w PMC11045866 38664378

[30] C. P. de Castro , B. M. Souza , P. Mancha‐Agresti , et al., “ *Lactococcus lactis* FNBPA+ (pValac: e6ag85a) Induces Cellular and Humoral Immune Responses After Oral Immunization of Mice,” Frontiers in Microbiology 12 (2021): 676172. 34093498 10.3389/fmicb.2021.676172 PMC8173160

[31] D. P. Cook , C. Gysemans , and C. Mathieu , “ *Lactococcus lactis* as a Versatile Vehicle for Tolerogenic Immunotherapy,” Frontiers in Immunology 8 (2018): 1961. 29387056 10.3389/fimmu.2017.01961 PMC5776164

[32] F. D. Ihekweazu , M. A. Engevik , W. Ruan , et al., “ *Bacteroides ovatus* Promotes IL‐22 Production and Reduces Trinitrobenzene Sulfonic Acid–Driven Colonic Inflammation,” American Journal of Pathology 191, no. 4 (2021): 704–719. 33516788 10.1016/j.ajpath.2021.01.009 PMC8027925

[33] T.‐R. Wu , C.‐S. Lin , C.‐J. Chang , et al., “Gut Commensal *Parabacteroides goldsteinii* Plays a Predominant Role in the Anti‐Obesity Effects of Polysaccharides Isolated From Hirsutella Sinensis,” Gut 68, no. 2 (2019): 248–262. 30007918 10.1136/gutjnl-2017-315458

[34] C.‐H. Lai , T.‐L. Lin , M.‐Z. Huang , et al., “Gut Commensal Parabacteroides Goldsteinii MTS01 Alters Gut Microbiota Composition and Reduces Cholesterol to Mitigate Helicobacter Pylori‐Induced Pathogenesis,” Frontiers in Immunology 13 (2022): 916848. 35844600 10.3389/fimmu.2022.916848 PMC9281563

[35] J. Fu , G. Li , X. Li , et al., “Gut Commensal Alistipes as a Potential Pathogenic Factor in Colorectal Cancer,” Discover Oncology 15, no. 1 (2024): 473. 39331213 10.1007/s12672-024-01393-3 PMC11436608

[36] J. J. Kopper , K. R. Theis , N. I. Barbu , et al., “Comparison of Effects of Trichuris Muris and Spontaneous Colitis on the Proximal Colon Microbiota in C3H/HeJ and C3Bir IL10−/−Mice,” Comparative Medicine 71, no. 1 (2021): 46–65. 33334395 10.30802/AALAS-CM-20-000021 PMC7898169

[37] J. B. Holm , D. Sorobetea , P. Kiilerich , et al., “Chronic Trichuris Muris Infection Decreases Diversity of the Intestinal Microbiota and Concomitantly Increases the Abundance of Lactobacilli,” PLoS One 10, no. 5 (2015): e0125495. 25942314 10.1371/journal.pone.0125495 PMC4420551

[38] Z. Wu , D. Pan , M. Jiang , L. Sang , and B. Chang , “Selenium‐Enriched Lactobacillus Acidophilus Ameliorates Dextran Sulfate Sodium‐Induced Chronic Colitis in Mice by Regulating Inflammatory Cytokines and Intestinal Microbiota,” Frontiers in Medicine 8 (2021): 716816. 34532332 10.3389/fmed.2021.716816 PMC8439139

[39] A. Bircher , E. Katkeviciute , Y. Morsy , S. Lang , A. Montalban‐Arques , and M. Scharl , “ *Roseburia intestinalis* Modulates Immune Responses by Inducing M1 Macrophage Polarization,” International Journal of Molecular Sciences 26, no. 11 (2025): 5049. 40507861 10.3390/ijms26115049 PMC12155563

[40] W. Luo , Z. Shen , M. Deng , et al., “ *Roseburia intestinalis* Supernatant Ameliorates Colitis Induced in Mice by Regulating the Immune Response,” Molecular Medicine Reports 20, no. 2 (2019): 1007–1016. 31173202 10.3892/mmr.2019.10327 PMC6625378

[41] Z. Shen , W. Luo , B. Tan , et al., “ *Roseburia intestinalis* Stimulates TLR5‐Dependent Intestinal Immunity Against Crohn's Disease,” eBioMedicine 85 (2022): 104285. 36182776 10.1016/j.ebiom.2022.104285 PMC9526137

[42] S. Bamba , O. Inatomi , A. Nishida , et al., “Relationship Between the Gut Microbiota and Bile Acid Composition in the Ileal Mucosa of Crohn's Disease,” Intestinal Research 20, no. 20 (2022): 370–380. 33975420 10.5217/ir.2021.00054 PMC9344239

[43] H. Sun , X. Su , Y. Liu , G. Li , and Q. Du , “ *Roseburia intestinalis* Relieves Intrahepatic Cholestasis of Pregnancy Through Bile Acid/FXR‐FGF15 in Rats,” Iscience 26, no. 12 (2023): 108392. 38025767 10.1016/j.isci.2023.108392 PMC10679810

[44] J. R. Caso , K. S. MacDowell , A. González‐Pinto , et al., “Gut Microbiota, Innate Immune Pathways, and Inflammatory Control Mechanisms in Patients With Major Depressive Disorder,” Translational Psychiatry 11, no. 1 (2021): 645. 34934041 10.1038/s41398-021-01755-3 PMC8692500

[45] B. Maldonado‐Arriaga , S. Sandoval‐Jiménez , J. Rodríguez‐Silverio , et al., “Gut Dysbiosis and Clinical Phases of Pancolitis in Patients With Ulcerative Colitis,” Microbiology 10, no. 2 (2021): e1181. 10.1002/mbo3.1181 PMC8087925 33970546

[46] S. B. Singh , A. Carroll‐Portillo , and H. C. Lin , “Desulfovibrio in the Gut: The Enemy Within?,” Microorganisms 11, no. 7 (2023): 1772. 37512944 10.3390/microorganisms11071772 PMC10383351

[47] G. Huang , Y. Zheng , N. Zhang , et al., “ *Desulfovibrio vulgaris* Caused Gut Inflammation and Aggravated DSS‐Induced Colitis in C57BL/6 Mice Model,” Gut Pathogens 16, no. 1 (2024): 39. 39060944 10.1186/s13099-024-00632-w PMC11282857

[48] W. Cao , R. W. Li , Y. Chin , Y. Wang , C. Xue , and Q. Tang , “Transcriptome Analysis Reveals the Protective Role of Fructo‐Oligosaccharide in Colonic Mucosal Barriers in Exercise‐Induced Stressed Mice,” Food & Function 12, no. 10 (2021): 4484–4495. 33885098 10.1039/d0fo02556a

[49] L. Xiao , B. Chen , D. Feng , T. Yang , T. Li , and J. Chen , “TLR4 May be Involved in the Regulation of Colonic Mucosal Microbiota by Vitamin A,” Frontiers in Microbiology 10 (2019): 268. 30873131 10.3389/fmicb.2019.00268 PMC6401601

[50] G. den Besten , K. van Eunen , A. K. Groen , K. Venema , D.‐J. Reijngoud , and B. M. Bakker , “The Role of Short‐Chain Fatty Acids in the Interplay Between Diet, Gut Microbiota, and Host Energy Metabolism,” Journal of Lipid Research 54, no. 9 (2013): 2325–2340. 23821742 10.1194/jlr.R036012 PMC3735932

[51] W. M. de Vos , H. Tilg , M. Van Hul , and P. D. Cani , “Gut Microbiome and Health: Mechanistic Insights,” Gut 71, no. 5 (2022): 1020–1032. 35105664 10.1136/gutjnl-2021-326789 PMC8995832

[52] E. P. J. G. Neis , C. H. C. Dejong , and S. S. Rensen , “The Role of Microbial Amino Acid Metabolism in Host Metabolism,” Nutrients 7, no. 4 (2015): 2930–2946. 25894657 10.3390/nu7042930 PMC4425181

[53] Z.‐N. Ling , Y.‐F. Jiang , J.‐N. Ru , J.‐H. Lu , B. Ding , and J. Wu , “Amino Acid Metabolism in Health and Disease,” Signal Transduction and Targeted Therapy 8, no. 1 (2023): 345. 37699892 10.1038/s41392-023-01569-3 PMC10497558

[54] L. F. Pedrosa , P. de Vos , and J. P. Fabi , “From Structure to Function: How Prebiotic Diversity Shapes Gut Integrity and Immune Balance,” Nutrients 16, no. 24 (2024): 4286. 39770907 10.3390/nu16244286 PMC11678351

[55] G. Liu , J. Tang , J. Zhou , and M. Dong , “Short‐Chain Fatty Acids Play a Positive Role in Colorectal Cancer,” Discover Oncology 15, no. 1 (2024): 425. 39256239 10.1007/s12672-024-01313-5 PMC11387572

[56] C. H. Kim , “Complex Regulatory Effects of Gut Microbial Short‐Chain Fatty Acids on Immune Tolerance and Autoimmunity,” Cellular & Molecular Immunology 20, no. 4 (2023): 341–350. 36854801 10.1038/s41423-023-00987-1 PMC10066346

[57] H. J. Flint , K. P. Scott , S. H. Duncan , P. Louis , and E. Forano , “Microbial Degradation of Complex Carbohydrates in the Gut,” Gut Microbes 3, no. 4 (2012): 289–306. 22572875 10.4161/gmic.19897 PMC3463488

