# Supplementary material for: The Influence of Innate Immunity, Adaptive Immunity and Diet on Intestinal Microbiota Following Trichuris muris Infection
Source: Parasite Immunol. 2026 Feb 2;48(2):e70060. doi: 10.1111/pim.70060 (PMC12865144; doi:10.1111/pim.70060)
Supplement: Supplementary file 1 — Figure S1: Gut microbiota composition across diet types. Stacked bar plots representing the relative abundance of bacterial genera across individual faecal samples. Taxa are shown at the genus level, with taxonomy annotated down to species where available. Each bar represents one sample, and colours correspond to distinct bacterial taxa, as indicated in the legend on the right. Figure S2: Comparison of alpha diversity in wild‐type WT mice fed either a ND or a HFD. (A) Shannon diversity index of faecal microbiota based on 16S rRNA gene sequencing, showing significantly reduced microbial diversity in WT mice fed a HFD (WT_H) compared to those on a ND (WT_N) (p = 0.0027, Wilcoxon test). (B) Shannon diversity index of caecal microbiota based on shotgun metagenomic sequencing. A trend toward reduced diversity in WT_HFD compared to WT_Normal was observed (p = 0.06, Wilcoxon test). These results suggest a consistent diet‐associated reduction in microbial diversity across different gut compartments and sequencing platforms. Figure S3: Gut microbial alpha diversity (Shannon index) in naive and infected mice across immune genotypes and dietary groups. (A–C) Comparisons of Shannon diversity in naive (uninfected) mice. (A) Alpha diversity by immune genotype (WT, RAG‐KO, RAGγc‐KO), regardless of diet. (B) Diversity stratified by both immune genotype and diet (ND vs. HFD). (C) Focused comparison within RAG‐KO naive mice, showing reduced diversity in mice on a HFD (RAG_H) versus ND (RAG_N). (D–F) Corresponding comparisons now including infected mice, to assess how Trichuris muris infection modifies gut microbial diversity across the same strata. (D) Diversity by genotype (infected vs. naive mice combined), (E) Stratified by both genotype and diet, (F) Focused comparison within RAG‐KO mice on HFD versus ND under infection. Figure S4: Microbial diversity (Shannon index) across different mouse genotypes under varying dietary and infection conditions. (A) Naive RAGγc‐KO, RAG‐KO, and W [file PIM-48-e70060-s001.docx]

**Supplementary**

**Supplementary figures**

|  |
| --- |
| ***Supplementary figure 1. Gut microbiota composition across diet types.*** *Stacked bar plots representing the relative abundance of bacterial genera across individual fecal samples. Taxa are shown at the genus level, with taxonomy annotated down to species where available. Each bar represents one sample, and colors correspond to distinct bacterial taxa, as indicated in the legend on the right.* |

|  |
| --- |
| ***Supplementary figure 2. Comparison of alpha diversity in wild-type WT mice fed either a ND or a HFD.*** ***(A)****Shannon diversity index of fecal microbiota based on 16S rRNA gene sequencing, showing significantly reduced microbial diversity in WT mice fed a HFD (WT_H) compared to those on a ND (WT_N) (*p*= 0.0027, Wilcoxon test).* ***(B)****Shannon diversity index of caecal microbiota based on shotgun metagenomic sequencing. A trend toward reduced diversity in WT_HFD compared to WT_Normal was observed (*p*= 0.06, Wilcoxon test). These results suggest a consistent diet-associated reduction in microbial diversity across different gut compartments and sequencing platforms.* |

|  |
| --- |
| ***Supplementary Figure3.*** ***Gut microbial alpha diversity (Shannon index) in naive and infected mice across immune genotypes and dietary groups.*** *(A–C) Comparisons of Shannon diversity in****naive (uninfected)****mice. (A) Alpha diversity by immune genotype (WT, RAG-KO, RAGγc-KO), regardless of diet. (B) Diversity stratified by both immune genotype and diet (ND vs HFD). (C) Focused comparison within RAG-KO naive mice, showing reduced diversity in mice on a HFD (RAG_H) versus ND (RAG_N). (D–F) Corresponding comparisons now including****infected mice,*** *to assess how*Trichuris muris*infection modifies gut microbial diversity across the same strata. (D) Diversity by genotype (infected vs naive mice combined), (E) Stratified by both genotype and diet, (F) Focused comparison within RAG-KO mice on HFD vs ND under infection.* |

| **** |
| --- |
| ***Supplementary Figure 4. Microbial diversity (Shannon index) across different mouse genotypes under varying dietary and infection conditions. (A)****Naive RAGγc-KO, RAG-KO, and WT mice on a ND, profiled using 16S rRNA sequencing of fecal samples. (****B)****Infected RAGγc-KO, RAG-KO, and WT mice on a ND, also assessed by 16S rRNA sequencing of fecal samples. (****C)****Infected RAGγc-KO, RAG-KO, and WT mice on a ND, profiled via shotgun metagenomics of fecal samples. (****D)****Naive RAGγc-KO, RAG-KO, and WT mice on a HFD, analyzed using 16S rRNA sequencing of fecal samples.*  ***(E)*** *Infected RAGγc-KO, RAG-KO, and WT mice on a HFD, with microbial diversity assessed from fecal samples using 16S rRNA sequencing.* ***(F)****Infected RAGγc-KO, RAG-KO, and WT mice on a HFD, profiled by shotgun metagenomics of caecal content. This comparative design highlights genotype-specific shifts in microbial diversity across dietary and infectious states, using both amplicon- and shotgun-based profiling approaches.* |

| **Comparison** | **statistic** | **p** | **p.adj** | **Significant** |
| --- | --- | --- | --- | --- |
| **RAG_H - RAG_N** | **3.40898485** | **0.000652051** | **0.00489038** | **TRUE** |
| **RAG_H - RAG/γC_H** | **0.32031354** | **0.748730657** | **0.80221142** | **FALSE** |
| **RAG_H - RAG/γC_N** | **2.55990433** | **0.010470098** | **0.02617525** | **TRUE** |
| **RAG_H - WT_H** | **-1.0187771** | **0.308308808** | **0.35574093** | **FALSE** |
| **RAG_H - WT_N** | **2.44951099** | **0.014305035** | **0.03065365** | **TRUE** |
| **RAG_N - RAG/γC_H** | **-3.2184929** | **0.001288662** | **0.00512018** | **TRUE** |
| **RAG_N - RAG/γC_N** | **-1.2258911** | **0.220239657** | **0.3003268** | **FALSE** |
| **RAG_N - WT_H** | **-3.9247915** | **8.6804900010372e-05** | **0.00130207** | **TRUE** |
| **RAG_N - WT_N** | **-1.0470874** | **0.295059283** | **0.35574093** | **FALSE** |
| **RAG/γC_H - RAG/γC_N** | **2.32354428** | **0.020149932** | **0.03778112** | **TRUE** |
| **RAG/γC_H - WT_H** | **-1.3134659** | **0.18902599** | **0.28353898** | **FALSE** |
| **RAG/γC_H - WT_N** | **2.22511156** | **0.02607375** | **0.04345625** | **TRUE** |
| **RAG/γC_N - WT_H** | **-3.2018708** | **0.001365382** | **0.00512018** | **TRUE** |
| **RAG/γC_N - WT_N** | **0.11264874** | **0.910309039** | **0.91030904** | **FALSE** |
| **WT_H - WT_N** | **3.09000609** | **0.002001524** | **0.00600457** | **TRUE** |

***Supplementary Table 1.*** Pairwise post-hoc comparisons of intestinal worm burdens across genotype–diet groups.
Dunn’s post-hoc tests were performed following a significant Kruskal–Wallis test (p = 5.8 × 10⁻⁵) to assess pairwise differences in worm counts between wild-type (WT), RAG-deficient (RAG), and RAGγc-deficient (RAGγc) mice fed either a normal diet (ND) or high-fat diet (HFD). The table reports the test statistic, unadjusted p-values, Benjamini–Hochberg–adjusted p-values (p.adj), and significance calls. Significant comparisons (p.adj < 0.05) highlight both genotype-dependent effects (e.g., impaired parasite control in RAG mice) and diet-specific effects within genotypes (e.g., improved clearance in WT mice on HFD).

**Supplementary methods**

**Parasite-specific Antibody ELISA**

Systemic humoral responses to T. muris infection were assessed by quantifying parasite-specific IgG1 and IgG2a/c antibodies in mouse serum using enzyme-linked immunosorbent assay (ELISA). High-binding 96-well plates (NUNC MaxiSorp) were coated overnight at 4°C with T. muris excretory/secretory (E/S) antigen at a concentration of 5 μg/mL in 0.05 M carbonate-bicarbonate buffer (pH 9.6). E/S antigen was prepared from adult-stage T. muris worms collected from infected mice at day 42 post-infection. Plates were washed five times with phosphate-buffered saline containing 0.05% Tween-20 (PBS-T) and blocked with 3% bovine serum albumin (BSA) in PBS for 1 hour at room temperature.

Serum samples were initially diluted 1:20 in PBS-T, followed by serial two-fold dilutions. Fifty microliters of diluted serum were added per well and incubated for 1 hour at room temperature. After washing, 50 μL of biotinylated rat anti-mouse IgG1 (Bio-Rad MCA336B, 1:2000) or IgG2a/c (BD 553388, 1:1000) diluted in PBS-T were added to the appropriate wells and incubated for 1 hour. Plates were washed again and incubated with 75 μL of streptavidin–horseradish peroxidase (HRP) (Roche, 1:1000 dilution) for 1 hour at room temperature.

Colour development was achieved using ABTS substrate, freshly prepared by mixing 1 mL ABTS stock (0.5 g ABTS in 50 mL citrate buffer) with 9 mL citrate buffer and 1 μL of 30% hydrogen peroxide. One hundred microliters of substrate were added to each well, and absorbance was read at 405 nm (reference 490 nm) using a VersaMax microplate reader (Molecular Devices, UK). Antibody responses were plotted as optical density (OD) values across serial serum dilutions.

**Microbiome Sample Preparation and Sequencing**

Microbial DNA was extracted from caecal and faecal samples using the QIAamp DNA Stool Mini Kit (Qiagen) according to the manufacturer’s instructions. The V3–V4 hypervariable region of the 16S rRNA gene was amplified using the Illumina 16S Metagenomic Sequencing Library Preparation protocol (Part # 15044223 Rev. B).

In brief, the first PCR used universal primers targeting the V3–V4 region (forward primer: 341F 5’-CCTACGGGNGGCWGCAG-3’; reverse primer: 806R 5’-GACTACHVGGGTATCTAATCC-3’) with Illumina overhang adapters. PCR cycling conditions were as follows: initial denaturation at 95°C for 3 minutes, followed by 25 cycles of denaturation at 95°C for 30 seconds, annealing at 55°C for 30 seconds, extension at 72°C for 30 seconds, and a final extension at 72°C for 5 minutes.

PCR amplicons were cleaned using AMPure XP magnetic beads (Beckman Coulter). Index PCR was performed using Nextera XT dual indices under the following conditions: initial denaturation at 95°C for 3 minutes, 8 cycles of denaturation at 95°C for 30 seconds, annealing at 55°C for 30 seconds, extension at 72°C for 30 seconds, and a final extension at 72°C for 5 minutes. Libraries were purified again with AMPure XP beads, quantified using Qubit dsDNA HS Assay (Thermo Fisher Scientific), and normalized to a final concentration of 4 nM. Libraries were pooled, denatured, and sequenced on an Illumina MiSeq platform using a 2 × 300 bp paired-end MiSeq Reagent Kit v3 (600-cycle) at the Bioinformatics Core Facility, University of Manchester. Caecal contents were subjected to shotgun metagenomic sequencing. DNA was extracted using standardized protocols, and libraries were prepared and sequenced by Transnetyx (UK; [https://www.transnetyx.com](https://www.transnetyx.com/)) using Illumina short-read technology. Quality control, host DNA removal, and taxonomic profiling were performed using standard bioinformatic pipelines.

**Microbiome Bioinformatics Processing**

Raw demultiplexed FASTQ files were processed using QIIME2 (version 2023.2). Primers were removed using Cutadapt (version 4.4) with default parameters. Reads were denoised, quality filtered, merged, and checked for chimeras using DADA2 (implemented within QIIME2) to generate amplicon sequence variants (ASVs) with high-resolution taxonomic specificity. ASVs were classified against the Greengenes 13.8 reference database using a Naive Bayes classifier.

To standardize sampling depth, rarefaction was performed to 20,000 sequences per sample. Microbial alpha diversity was assessed using the Shannon diversity index and observed richness. **Beta diversity** was calculated using Bray–Curtis dissimilarity matrices to assess differences in overall microbial community composition between groups. These differences were visualized using Principal Coordinate Analysis (PCoA) plots, which display the major axes of variation in microbial profiles across samples. Statistical significance of group-level differences in beta diversity was tested using permutational multivariate analysis of variance (PERMANOVA) with 999 permutations. To identify microbial taxa differentially abundant across experimental groups, we performed differential abundance (DA) testing using the DESeq2 method implemented within the phyloseq and DESeq2 packages in R. A Wald test was used to estimate log₂ fold changes in microbial abundance between groups. Resulting p-values were corrected for multiple testing using the Benjamini–Hochberg false discovery rate (FDR) procedure. Taxa with an FDR-adjusted p < 0.05 were considered statistically significant. To visualize differentially abundant taxa, we generated volcano plots (log₂ fold change vs. –log₁₀ FDR-adjusted p-value) and waterfall plots. In the latter, we ranked significantly differentially abundant taxa by descending absolute log₂ fold change, using a cutoff of |log₂ fold change| ≥ 1 to highlight the most strongly altered microbes.
